# Development of a continuous process for α-thio-β-chloroacrylamide synthesis with enhanced control of a cascade transformation

**DOI:** 10.3762/bjoc.12.246

**Published:** 2016-11-24

**Authors:** Olga C Dennehy, Valérie M Y Cacheux, Benjamin J Deadman, Denis Lynch, Stuart G Collins, Humphrey A Moynihan, Anita R Maguire

**Affiliations:** 1 Department of Chemistry, Analytical and Biological Chemistry Research Facility, Synthesis and Solid State Pharmaceutical Centre, University College Cork, Cork, Ireland; 2 Department of Chemistry and School of Pharmacy, Analytical and Biological Chemistry Research Facility, Synthesis and Solid State Pharmaceutical Centre, University College Cork, Cork, Ireland

**Keywords:** α-thio-β-chloroacrylamides, cascade reactions, flow chemistry

## Abstract

A continuous process strategy has been developed for the preparation of α-thio-β-chloroacrylamides, a class of highly versatile synthetic intermediates. Flow platforms to generate the α-chloroamide and α-thioamide precursors were successfully adopted, progressing from the previously employed batch chemistry, and in both instances afford a readily scalable methodology. The implementation of the key α-thio-β-chloroacrylamide casade as a continuous flow reaction on a multi-gram scale is described, while the tuneable nature of the cascade, facilitated by continuous processing, is highlighted by selective generation of established intermediates and byproducts.

## Introduction

**Scheme 1:**
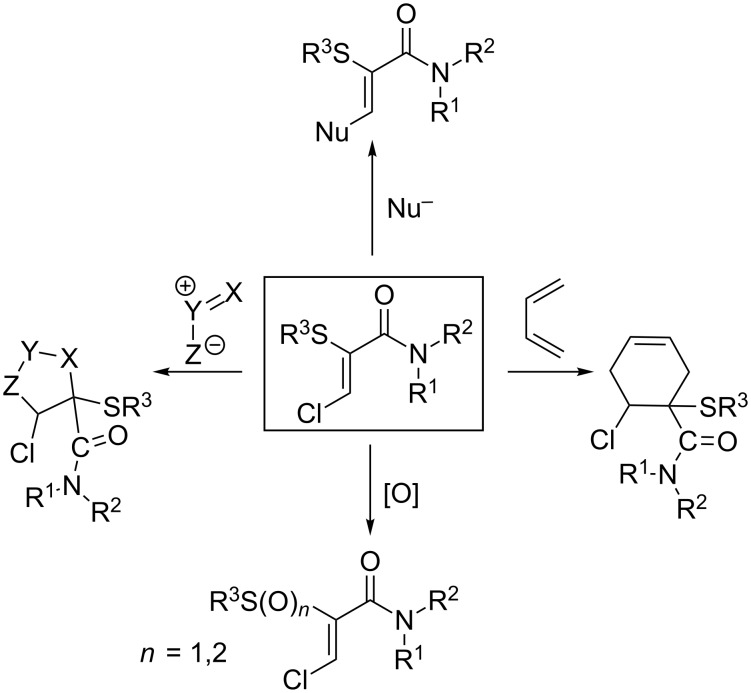
Reaction pathways of α-thio-β-chloroacrylamides.

Since the efficient and highly stereoselective transformation of α-thioamides to the corresponding α-thio-β-chloroacrylamides derivatives was first reported [1–2], the considerable synthetic utility of these heavily functionalized acrylamide compounds has been well documented [3]. The predominant site of reactivity is at the electrophilic β-carbon, which results from the combined influence of the amide and chloro substituents, mitigating the electron-donating effect of the sulfide moiety. Nucleophilic substitution [4], Diels–Alder reactions [5] and 1,3-dipolar cycloadditions [6–9], and oxidation of the sulfide group [10–12] are among a wide array of transformations which have been successfully applied to these compounds (Scheme 1).

**Scheme 2:**
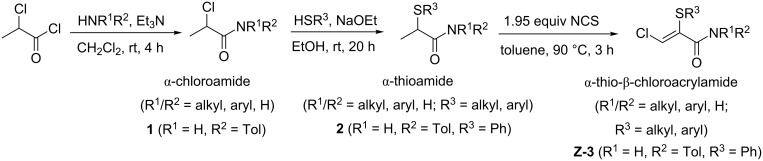
Typical three-step batch preparation of α-thio-β-chloroacrylamide.

In order to fully exploit the synthetic potential of these β-chloroacrylamides, however, a means of ready access to appreciable quantities of material is required. Preparation of α-thio-β-chloroacrylamides typically results from a three-step synthetic route, culminating in a final cascade/domino reaction [13] where a toluene solution of α-thioamide and NCS is subjected to a ‘hot plunge’ by placing it into an oil bath at 90 °C (Scheme 2). While this route has consistently provided a robust means of generating the desired β-chloroacrylamides at scales of 1–10 g, it suffers from several disadvantages which impact on the ease of scale-up.

The preparation of the α-chloroamide **1** is exothermic and requires significant external cooling, an undesirable feature for scale-up. The synthesis of the α-thioamide **2** involves prior generation of fresh sodium ethoxide from sodium metal. Furthermore, this α-thioamide protocol, at high pH, ordinarily does not go to completion, leaving unreacted starting material and forming impurities which are subsequently removed by chromatographic purification. Finally, the optimized conditions for the final cascade transformation employ rapid heating via ‘hot-plunge’ in order to minimize the formation of process impurities during the initial heating phase [1]. This efficient rapid heating poses practical difficulties for scale-up and, furthermore, chromatographic separation is required to remove product impurities.

The nature of the aforementioned difficulties outlined are, however, largely specific to the scale-up of batch chemistry. A continuous processing approach frequently possesses advantages over the batch equivalent, as has been extensively documented [14–22]. When combined with automated operation, it allows for enhanced reproducibility and access to extreme conditions, which, along with improved heat and mass transfer, all facilitate significant ease of scale-up. The reaction control afforded by use of high surface-area-to-volume ratio tubular reactors, specifically with respect to dissipation of heat, offers a safety profile unique to flow chemistry. Continuous processing also provides the capacity to continuously generate hazardous reagents and intermediates in small quantities, in situ, and transferred directly into a reaction stage without operator handling [21–26]. As rapid heat transfer (steps 1 and 3) and greater reaction control (steps 2 and 3) were identified as the key challenges to be overcome, we envisaged that continuous processing could facilitate the preparation of large quantities of α-thio-β-chloroacrylamide with reduced purification requirements. The goal of this study was to develop an optimized process for the synthesis of α-thio-β-chloroacrylamides, employing a model system with *N*-4′-methylphenyl-(*Z*)-3-chloro-2-(phenylthio)propenamide (*Z-***3**) as the target product. This optimized process would utilise flow chemistry as a key enabling technology to overcome the aforementioned challenges.

## Results and Discussion

### Preparation of *α*-chloroamide

**Scheme 3:**
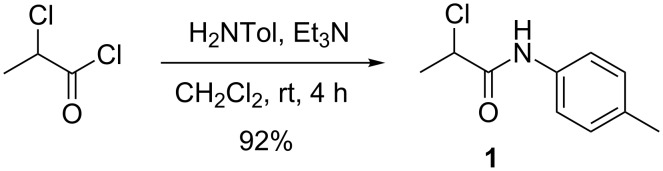
Batch process for preparation of α-chloroamide **1**.

The synthesis of α-chloroamide **1** is highly exothermic, due to the neutralisation of HCl – a byproduct – with triethylamine, and the need for effective heat removal imposes limitations on the batch scale-up of this step. It was envisaged that the efficient heat transfer properties of a high surface area tubular flow reactor would remove the need for external cooling of the reaction. To facilitate safe scale-up of this reaction we initially investigated a direct transfer of the batch process (Scheme 3) to continuous mode.

**Scheme 4:**
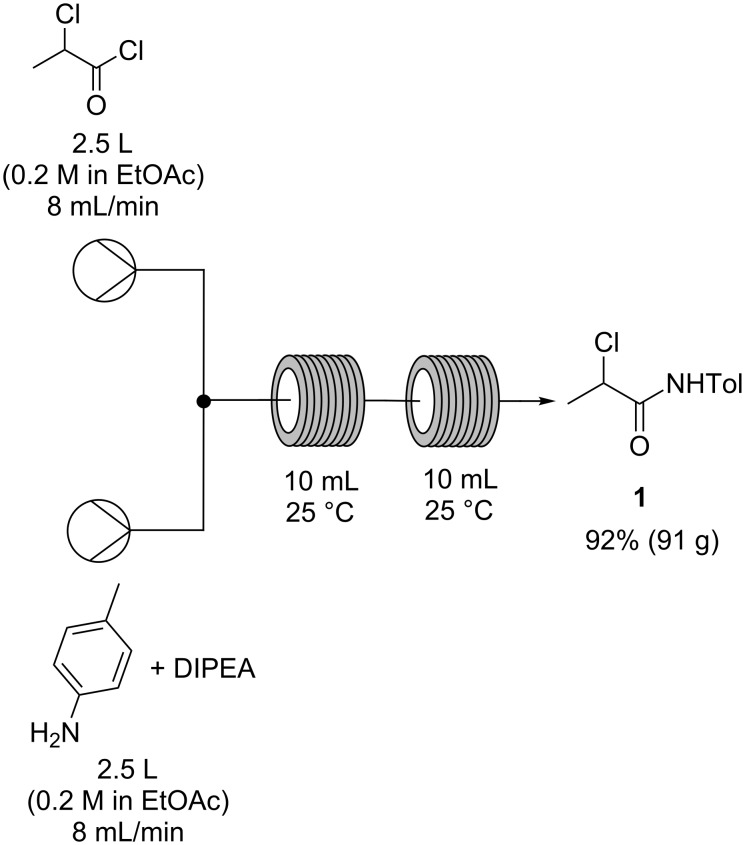
Process for the conversion of 2-chloropropionyl chloride and *p*-toluidine to α-chloroamide **1** under optimized flow conditions.

Although initial investigations involving small throughput had shown promise, the practicalities of employing dichloromethane at process scales caused us to consider alternative ‘greener’ solvent systems [27]. A screen of alternative solvents in batch test reactions revealed that, while the amide formation was tolerant to most solvents, rapid precipitation of triethylamine hydrochloride would be problematic in a continuous process. Indeed, trial runs of a continuous process in ethyl acetate resulted in immediate blockage of the flow reactor at the point of reagent mixing. To prevent blockages due to salt formation we investigated replacements for triethylamine that would produce a more soluble HCl salt. Diisopropylethylamine (DIPEA) was found to be a suitable base that allowed the continuous process to be carried out in ethyl acetate without any observed precipitation of the HCl salt. The ‘greener’ continuous amide formation (Scheme 4) was carried out on a large scale, producing 91 g (92% yield) of the α-chloroamide **1** over 5 hours of continuous operation, as a white crystalline solid after aqueous work-up and recrystallization.

### Synthesis of α-thioamide

Driving the reaction to completion and avoiding the use of sodium metal were the key aims in transferring α-thioamide preparation from batch to flow. Although yields of 80–90% can be obtained under batch conditions, incomplete conversion to α-thioamide **2** necessitates a difficult, and often laborious, chromatographic separation, as starting material **1** and product **2** are poorly resolved. Indeed, high-purity batches of α-thioamide **2** are often not achieved by chromatography, with the resulting product typically ca. 94% pure by HPLC. It was also envisaged that the facility to superheat the solvent in a pressurised continuous platform could enable sodium ethoxide to be replaced by a weaker base, obviating the need for sodium metal.

At an early stage of process development, the possibility of telescoping the amide formation and thiolation steps was considered. Attempts were made to use triethylamine as the base in a continuous thiolation reaction, however, the reaction was found to progress slowly and a maximum conversion of 39% was observed by ^1^H NMR spectroscopy. Elevating the temperature to 90 °C and employing DBU, as a more basic alternative to triethylamine, did not increase the reactivity. Hence, the focus was instead directed on converting the existing batch process (Scheme 2), with sodium ethoxide as base, into a stand-alone continuous process.

**Scheme 5:**
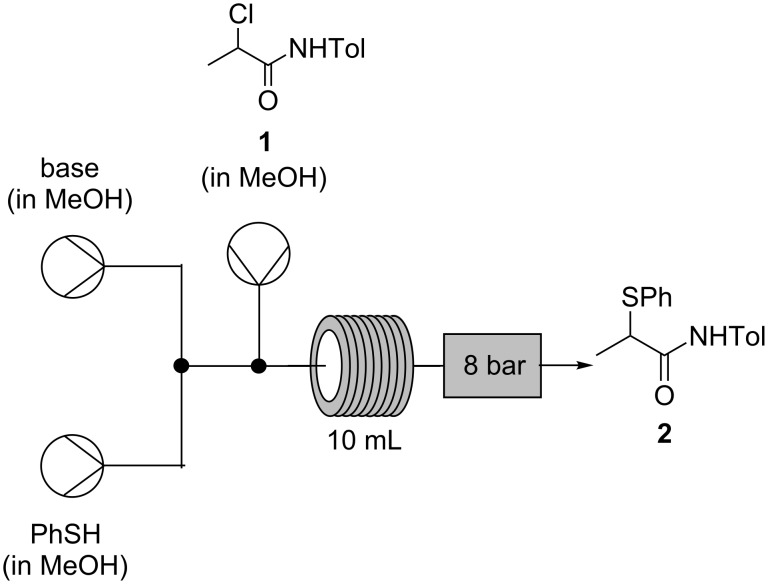
Conversion of **1** to **2** in continuous mode using MeOH as solvent.

Initially, however, the sodium chloride byproduct was found to precipitate from ethanol causing blockages at the back-pressure regulator. As sodium chloride possesses a relatively low solubility in ethanol (ca. 0.055 g in 100 g of ethanol at 20 °C) compared to methanol (1.375 g in 100 g) [28], methanol was proposed as an alternative solvent. As ^1^H NMR analysis indicated that the crude reaction product from batch tests (using methanol as solvent) consisted of 98% α-thioamide **2**, the process was subsequently transferred to a continuous flow system (Scheme 5).

A variety of temperatures (60–120 °C), bases (NaOMe, NaOH, Na_2_CO_3_) and concentrations (0.1–0.3 M) were investigated using methanol as solvent (see Supporting Information File 1), however, unreacted *α*-chloroamide **1** and diphenyl disulfide were detected as product components in all experiments. Direct sampling of the reaction mixture (system effluents) also showed additional component peaks by HPLC analysis, which were not observed in material isolated after the reaction work-up. While temperatures above 100 °C or α-chloroamide **1** concentrations above 0.1 M were not found to be advantageous, sodium hydroxide demonstrated promising results when used as base.

With use of sodium hydroxide in mind, replacement of methanol with an ethanol/water mixture as solvent was subsequently examined. This solvent change was investigated in conjunction with further refinements to the stoichiometry of sodium hydroxide and thiophenol used, along with optimization of process temperature and residence time (Table 1).

**Table 1:** Optimization of temperature, thiophenol concentration, residence time and stoichiometry of base for conversion of **1** to **2** in continuous mode^a^ using EtOH/H_2_O as solvent.

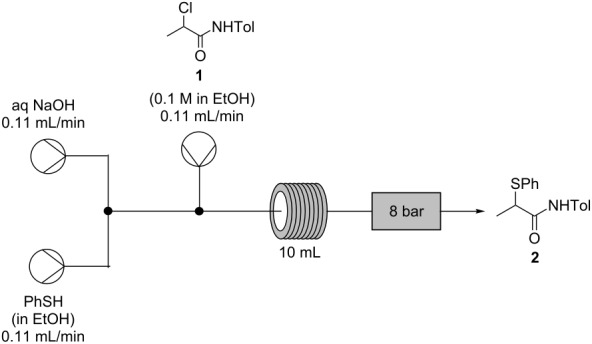								

Entry	Residence time (min)	Temp. (°C)	PhSH (equiv)	NaOH (equiv)	Product ratio			
					**2** (%)^b^	**1** (%)^b^	PhSSPh (%)^b^	Other^c^ (%)^b^

1	30	100	1.4	10	73.2	0	2.4	24.4
2	30	120	1.4	10	82.2	0	6.1	11.7
3	30	140	1.4	10	54.5	0	1.3	44.2
4	30	100	1.4	5	75.9	0	2.6	21.5
5	30	100	1.2	5	78.8	3.6	3.5	14.2
6	30	100	1.1	5	81.0	0.4	0.6	18.0
7	30	100	1.05	5	85.4	0	1.1	13.5
8	10	100	1.05	5	67.4	8.5	0.4	23.7
9	10	120	1.05	5	77.3	0	0.9	21.8
10	5	120	1.05	5	81.0	2.1	1.2	15.7
11	2	120	1.05	5	72.8	4.5	1.1	21.6
12	2	140	1.05	5	71.3	0	1.3	27.4
13^d^	5	120	1.05	5	74.1	0	0	25.9

^a^General conditions: 1 equiv α-chloroamide **1** (2 mL of a 0.1 M solution in EtOH) was reacted with PhSH (as a solution in EtOH) and NaOH (as a solution in H_2_O). ^b^Determined by HPLC analysis (peak area: see Supporting Information File 1) of samples taken directly from flow reactor as effluent solutions and diluted in MeCN prior to analysis. ^c^Unisolated components, not present after work-up. ^d^Reaction was run using 2 mL 0.25 M solution of α-chloroamide **1** in EtOH.

Initially, when using 10 equivalents of sodium hydroxide, the best conversion to product **2** was obtained at a reaction temperature of 120 °C (entry 2, Table 1), with no unreacted α-chloroamide **1** detected by HPLC. Employing just 5 equivalents of hydroxide also provided an acceptable yield of α-thioamide **2** in all instances (entries 4–13, Table 1). The use of an excess of sodium hydroxide as base had removed the difficulty with unreacted starting material, presumably by hydrolysis of unreacted α-chloroamide **1** to more water soluble byproducts. In order to minimize the presence of diphenyl disulfide in the isolated product, the stoichiometry of thiophenol was also examined. Interestingly, a reduction in the excess of thiophenol to 1.05 equivalents was found to give a greater proportion of α-thioamide **2** and significantly reduced level of diphenyl disulfide (entries 7–13, Table 1).

After an improved stoichiometry of reagents had been established, lowering the residence time was investigated to facilitate efficient large scale synthesis by a continuous flow process. Ultimately, a residence time of 5 min at 120 °C, using a 0.25 M concentration of α-chloroacrylamide, was found to give an acceptable quality of product **2**, with no detectable quantities of starting material **1** or diphenyl disulfide by HPLC analysis (entry 13, Table 1).

**Scheme 6:**
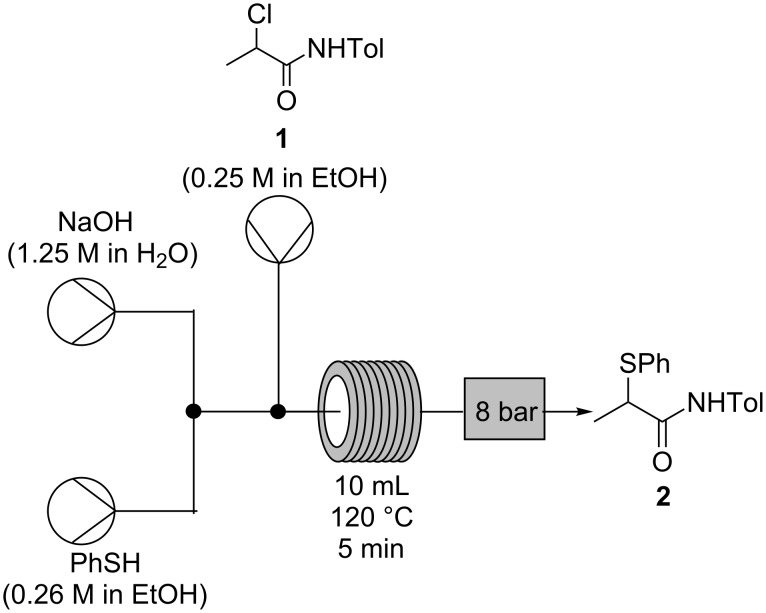
Optimized process for the conversion of α-chloroamide **1** to α-thioamide **2** under flow conditions.

The optimized continuous process (Scheme 6) was then run on a 5 g scale with no observed loss of yield or purity. The α-thioamide **2**, which crystallized directly from the output of the flow process, was obtained in 71% yield and found to be >99% pure by HPLC analysis, compared to 94% purity for a typical batch preparation following chromatography.

As transferring the α-thioamide preparation to a continuous platform had involved a number of important changes to the reaction conditions, it was decided to evaluate the optimized flow conditions (entry 13, Table 1) when applied to a batch process for comparison: 1.05 equivalents of thiophenol in ethanol mixed with an aqueous solution containing 5 equivalents of sodium hydroxide followed by heating to reflux for 1 hour. Initially on a 500 mg scale, a 97% yield of α-thioamide **2** was obtained, while operating at a higher concentration (increased from 0.25 M α-chloroamide **1** to 0.4 M α-chloroamide **1**) a yield of 94% was achieved on a 5 g scale, with the isolated product determined to be 99% pure by HPLC analysis. This process was ultimately carried out at a 20 g scale achieving 88% yield, again with 99% purity; the decrease in yield was offset by the increase in both productivity at this scale and product purity. The ability to operate effectively at higher concentrations in batch than in flow, in this case, made this batch process the optimum method of α-thioamide preparation, with a considerable reduction in reaction time from 10 hours to just 1 hour (for 20 g of **2**) and with a reduction of approximately one third in the required solvent volume, compared to the flow process. By comparison, the original batch process was typically run for 20 hours on scales up to 10 g.

As with the optimized flow process, direct crystallisation of the α-thioamide product **2** from this improved batch process was achieved by cooling and adding water as anti-solvent. This method of product isolation obviated the need for the arduous work-up – involving extraction into dichloromethane and several aqueous washes – associated with the original batch version, and gave material which was 99% purity or greater by HPLC analysis.

The stoichiometry of sodium hydroxide required for reaction completion was also considered as part of the batch comparison. Here, a reduction from 5 equivalents to 3 and subsequently to just 2 equivalents was found to be possible, with no discernible negative impact on the product formation. In the latter case, in batch the α-thioamide **2** was recovered in 92% yield and >99% purity by HPLC, when the reaction was performed on a 5 g scale. A subsequent batch run on a 20 g afforded an 89% yield, with the same level of product purity. It is, perhaps, worth noting that the high isolated yields obtained from the scaled-up reactions strongly suggest that the substantial quantities of ‘other’ components observed by HPLC analysis, but removed during work-up, are overestimated by detection at 250 nm (Table 1). Such an overestimation is consistent with the presence of additional chromophores, when compared to the desired product, and would indicate that these observed components may contain an α,β-unsaturated carbonyl motif in their structures.

The value of exploring flow methodology, ultimately leading to an improved batch process, is keenly highlighted in this instance. The optimized batch process, developed through examining the use of continuous processing, can produce 20 g of pure material, with direct product precipitation/crystallization from the reaction solution (>99% pure by HPLC analysis), which has removed the requirements for isolation by extraction and subsequent chromatographic purification. HPLC analysis of the current process – in either batch or flow – indicated complete consumption of the α-chloroamide **1**, without diphenyl disulfide formation, while an increase in yield from 80–90% to consistently over 90% has been achieved. Furthermore, the use of an inert atmosphere is no longer necessary as the decrease in reaction time has essentially eliminated the opportunity for aerobic oxidation of the thiophenolate anion to diphenyl disulfide, while sodium metal is no longer used as part of the process.

### α-Thio-β-chloroacrylamide cascade in flow

**Scheme 7:**
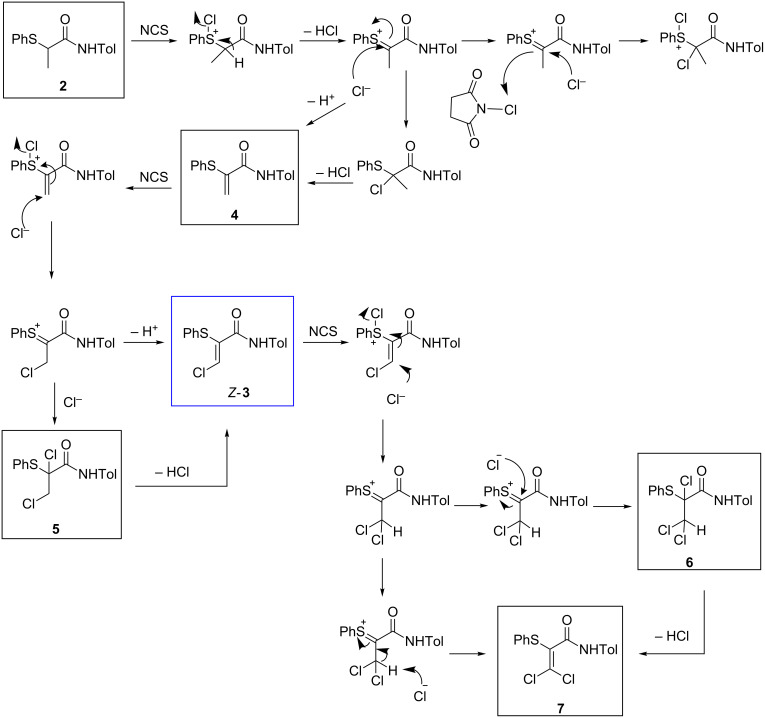
Mechanism of the β-chloroacrylamide cascade process [29].

Successful conversion of the β-chloroacrylamide cascade step from batch to flow posed a number of challenges. The reaction mechanism (Scheme 7) involves a complex cascade which also gives rise to several known impurities, including acrylamide **4**, dichloride **5**, trichloride **6** and dichloroacrylamide **7**.

In the optimized batch synthesis of α-thio-β-chloroacrylamide *Z-***3** from the corresponding α-thioamide **2**, *N*-chlorosuccinimide (NCS) is added in one portion to a solution of **2** in toluene and the reaction mixture is immediately immersed in an oil bath at 90 °C (Scheme 2). Although this protocol performs well, giving 91% yield on a ca. 5 g scale [1], the practical challenges of achieving efficient rapid heating on a larger scale in batch made continuous processing an attractive alternative for scale-up due to its capacity for excellent temperature control. Efficient heat transfer due to the high surface, low volume geometry of tubular flow reactors makes it possible to achieve extremely rapid temperature transitions. It was envisaged that flowing the reaction through a heated section of tubing would be analogous to the batch ‘hot plunge’ method but with the capacity for faster heating of the reaction.

Given the superior performance of α-thioamide **2** synthesis in batch, the potential telescoping of the thiolation process with the β-chloroacrylamide cascade was not investigated. Furthermore, the potential vulnerability of α-thio*-*β-chloroacrylamides towards nucleophilic substitution by an aqueous ethanol component of the reactant stream (from α-thioamide **2** preparation), particularly at elevated temperatures, strongly mitigated against integrating these steps.

For the batch process, the solubility of NCS in toluene has notable benefits: NCS is soluble in toluene at high temperatures, while the succinimide byproduct readily precipitates from toluene on cooling, allowing its convenient removal by filtration. In a continuous flow process, however, succinimide precipitation would cause blockage of the system.

Attempts at transferring the cascade reaction to a continuous platform began with direct adaptation of the existing batch process (Table 2). The solubility of NCS in toluene was found to be variable and often unsuitably low. Only batches of NCS which readily gave complete solutions were used and these batches were always either freshly recrystallized or commercial batches which were ‘newly’ opened prior to use. The reduced solubility of other batches was attributed to the partial hydrolysis of NCS upon intermittent exposure to ambient conditions over prolonged periods, also generating HCl.

**Table 2:** Initial flow process for conversion of **2** to *Z-***3** using toluene as solvent.

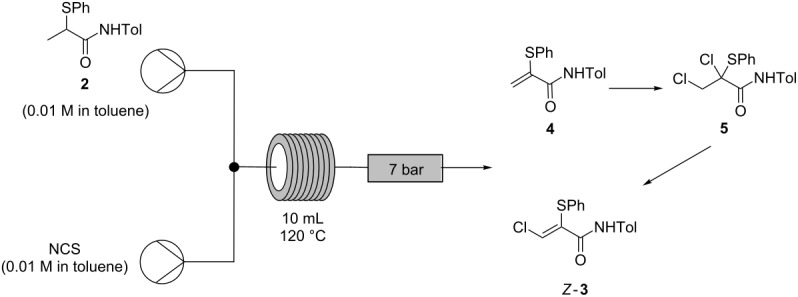						

Entry	Ratio **2**:NCS^a^	Residence Time (min)	Product ratio			
			**2** (%)^b^	**4** (%)^b^	**5** (%)^b^	*Z-***3** (%)^b^

1	1:2	20	19	15	18	47
2	1:2	50	21	19	0	60
3	1:2.3	20	22	8	20	46
4	1:3	20	25	1	12	62
5	1:1	20	21	77	<1	2

^a^Stoichiometric ratio of α-thioamide **2**:NCS controlled by manipulating the relative flow rates. ^b^Molar ratio determined by HPLC analysis (peak area weighted for relative response factors of each component: see Supporting Information File 1) of samples taken directly from flow reactor as effluent solutions and diluted in MeCN prior to analysis.

Initial investigations using our prototype flow process employed 0.01 M solutions of NCS and starting material **2** in toluene. The equivalent of a ‘hot-plunge’ method was achieved by passing the reaction solution through a coiled tube reactor at 120 °C. The high surface area–volume ratio of tubular flow reactors is ideal for such rapid temperature transitions. It was noted that a relatively short residence time of only 20 min could be used, with a longer time of 50 min offering only a modest improvement on the reaction outcome (entries 1 and 2, Table 2). Indeed, the conversion of starting material **2** to acrylamide **4** was found to be closely comparable, indicating almost identical reaction progress, given the instability of dichloride **5**, which easily converts to the final product *Z-***3**.

Increasing the amounts of NCS used was found to lead to a better conversion of acrylamide **4** to dichloride **5** or α-thio-β-chloroacrylamide *Z-***3** (entries 3 and 4, Table 2), with only 1% of acrylamide **4** left unreacted with three equivalents of NCS used. When only an exact stoichiometric ratio (1:1) of NCS was used, the reaction stopped after the first chlorination step, leading to a reaction mixture which contained acrylamide **4** as the main product formed (entry 5, Table 2). This ability to halt the cascade at the acrylamide intermediate **4** or push through to the α-thio-β-chloroacrylamide *Z*-**3** highlights the enhanced control of reaction stoichiometry afforded by a continuous platform and offers the possibility to isolate selected intermediates in the cascade reaction using a continuous process, more effectively than in batch and with greater flexibility.

### Optimization of the cascade process using flow chemistry

In all the aforementioned cases (Table 2), around 20% of the starting material was consistently found to be unreacted. The key limitation to overcome was proposed to be the low solubility of NCS in toluene, and the consequent limitations to reactor throughput. To offset this difficulty, the use of alternative solvents was investigated. Acetonitrile was considered as a possible alternative solvent due to the high solubility of NCS it offers. Hence, preliminary experiments were carried out in order to compare its performance to toluene (Table 3), with the α-thioamide **2**:NCS ratio again adjusted by manipulating the concentration of the reagent solutions. In these experiments, the reaction conversions were determined using ^1^H NMR analysis of the crude product material obtained, with characteristic proton signals of the β-carbon of the starting material **2**, intermediates **4** and **5**, and the desired product *Z-***3** being easily identifiable.

**Table 3:** Solvent screen for conversion of **2** to *Z-***3** in continuous mode.

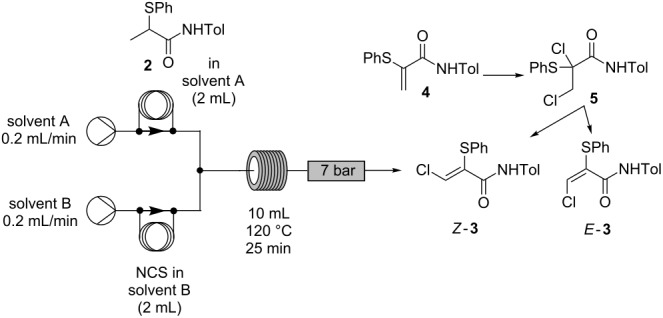								

Entry	[α-Thioamide **2**] (mM)	[NCS] (mM)	Solvent A/B	Product ratio				
				**2** (%)^a^	**4** (%)^a^	**5** (%)^a^	*Z-***3** (%)^a^	*E*-**3** (%)^a^

1	25	50	Tol/Tol	0	9.9	9.9	78.7	1.5
2	25	50	Tol/MeCN	0	4.3	0	81.4	14.3
3	25	50	MeCN/MeCN	0	0	0	86.9	13.1
4	200	400	Tol/MeCN	0	7.3	0	83.5	9.2
5	200	400	MeCN/MeCN	0	0	0	87.8	12.2

^a^Determined by ^1^H NMR spectroscopy.

Using toluene as a solvent for both reagents (α-thioamide **2** and NCS) leads to 10% unreacted acrylamide **4** (entry 1, Table 3). When a solution of **2** in toluene and a solution of NCS in acetonitrile were employed as the reactant streams, similar results were observed at either low or high concentration, in terms of residual acrylamide intermediate detected (entries 2 and 4, Table 3). However when acetonitrile was used as solvent for both reagents (α-thioamide **2** and NCS), full conversion to the final product *Z-***3** was observed, at both high and low concentration of reagents (entries 3 and 5, Table 3). Use of high concentrations has the advantage of increasing process productivity. In this case (entry 5, Table 3), the production could be increased eight-fold for the same reaction time as entry 3 (25 min residence time). Furthermore, higher concentration of reagents enables greener synthesis by reducing solvent use.

Interestingly, during development studies on the conversion of α-thioamide **2** to α-thio-β-chloroacrylamide Z-**3** in acetonitrile, by flow or in batch, a new component of the cascade reaction was observed, which was identified as the (*E*)-α-thio-β-chloroacrylamide *E-***3**.

An important feature of the experiments conducted on the β-chloroacrylamide cascade as a continuous process was the complete absence of the over-chlorinated products **6** and **7**, which were not observed by HPLC analysis or ^1^H NMR spectroscopy. In contrast, when similar conditions were employed in batch, significant formation of these byproducts was often in evidence [1]. The flow process for the conversion of α-thioamide **2** to α-thio-β-chloroacrylamide **3** which employed acetonitrile as solvent was therefore taken forward for optimization and scale-up (Table 4).

**Table 4:** Optimization of flow rates, residence time and temperature for conversion of **2** to *Z*-**3** in continuous mode^a^.

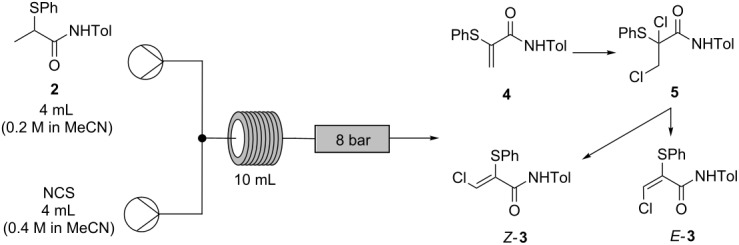							

Entry	Residence time (min)	Flow rate (mL/min)	Temp (°C)	Product ratio^b^			
				*Z-***3** (%)^c^	*E-***3** (%)^c^	**4** (%)^c^	**5** (%)^c^

1	25	0.2	120	88.2	11.8	0.0	0.0
2	15	0.3	120	87.6	12.4	0.0	0.0
3	10	0.5	120	86.9	13.1	0.0	0.0
4	5	1.0	120	85.5	14.5	0.0	0.0
5	2	2.5	120	72.8	13.7	0.0	13.5
6	2	2.5	80	4.9	2.3	0.0	92.7
7	2	2.5	90	9.5	3.1	0.0	87.4
8	2	2.5	100	17.3	4.4	0.0	78.3
9	2	2.5	130	84.0	16.0	0.0	0.0

^a^1 Equiv of α-thioamide **2** (4 mL of a 0.2 M solution in MeCN) was reacted with 2 equiv of NCS (4 mL of a 0.4 M solution in MeCN). ^b^Unisolated components, not present after work-up were not included, but ranged from 5–10% by peak area. ^c^Molar ratio determined by HPLC analysis (peak area weighted for relative response factors of each component: see Supporting Information File 1) of samples taken directly from flow reactor as effluent solutions and diluted in MeCN prior to analysis.

The residence time of the flow process was investigated to determine the completion time of the reaction, principally to minimize the extent of impurity formation due to over-reaction. The shortest possible effective residence time would be also preferable for larger scale operation in order to maximize the reactor throughput. The dichloride intermediate **5** was still present after a 2 min residence time (entry 5, Table 4), implying the reaction had not yet reached completion, while minor impurities remained at similar levels throughout all of the experiments. The succinimide byproduct was removed in the product work-up.

At lower reaction temperatures, large quantities of the dichloride **5** were observed (entries 6–8, Table 4), with correspondingly low quantities of product *Z-***3**. This finding is consistent with previous work showing that rapid heating resulted in a more efficient reaction cascade to the desired product *Z-***3**, while slower heating leads to substantial quantities of reaction intermediates **4** and **5** as product impurities [3].

The stoichiometry of NCS used for the continuous process was also further optimized (Table 5). It was found that, at 130 °C, 2 equivalents of NCS resulted in the lowest levels of the impurities arising from reaction intermediates and over-chlorination byproducts while also achieving one of the highest conversions to the desired α-thio-β-chloroacrylamide *Z*-**3** (82.9%, entry 4, Table 5).

**Table 5:** Optimization of NCS stoichiometry for conversion of α-thioamide **2** to α-thio-β-chloroacrylamide *Z-***3** in continuous mode^a^.

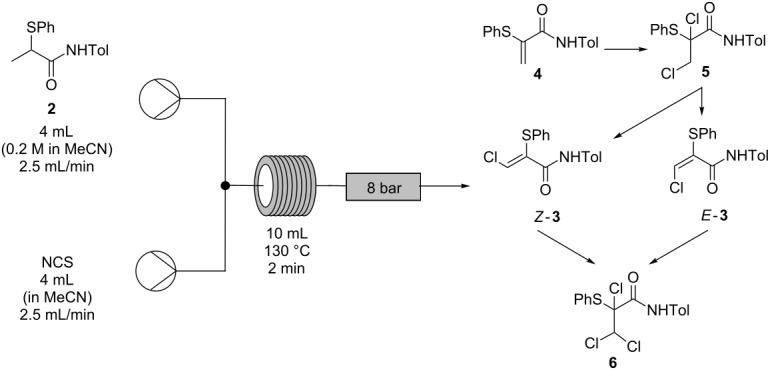						

Entry	NCS equiv	Product ratio^b^				
		*Z*-**3** (%)^c^	*E*-**3** (%)^c^	**4** (%)^c^	**5** (%)^c^	**6** (%)^c^

1	1.7	68.8	13.1	18.1	0.0	0.0
2	1.9	73.8	14.1	12.1	0.0	0.0
3	1.95	76.5	14.8	8.6	0.0	0.0
4	2	82.9	15.8	1.3	0.0	0.0
5	2.05	81.6	15.9	1.4	0.0	1.1
6	2.1	81.3	15.3	1.7	0.0	1.7
7	2.2	69.4	11.9	0.0	0.0	18.7

^a^1 Equiv of α-thioamide **2** (4 mL of 0.2 M solution in MeCN) was reacted with NCS (4 mL of solution in MeCN) at 130 °C for 2 min, using a flow rate of 2.5 mL/min. ^b^Molar ratio determined by HPLC analysis (peak area weighted for relative response factors of each component: see Supporting Information File 1) of samples taken directly from flow reactor as effluent solutions and diluted in MeCN prior to analysis. ^c^Unisolated components, not present after work-up were not included, but ranged from 2–14% by peak area.

**Scheme 8:**
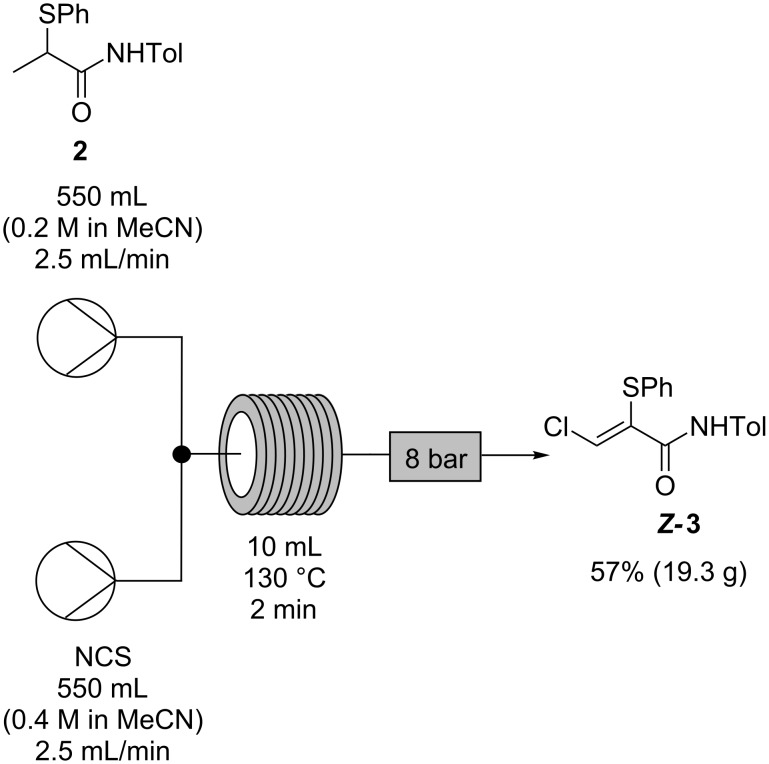
Optimized flow process for conversion of α-thioamide **2** to α-thio-β-chloroacrylamide *Z-***3**.

This process was then operated on a 30 g scale (Scheme 8) to produce 19.3 g (57% yield, >99% pure by HPLC analysis and ^1^H NMR spectroscopy) of isolated α-thio-β-chloroacrylamide *Z-***3** in less than 4 hours. The crude material was found to consist only of a mixture of the *Z*- and *E*-isomers by ^1^H NMR spectroscopy, with pure *Z*-**3** selectively recovered after recrystallization, albeit with a loss of isolated yield from this process. This is the first instance in which multi-gram quantities of the product *Z-***3** have been isolated without the need for chromatography and on more than 3 times the scale which can be obtained in batch with the same reaction time [1]; the increase in quantity and the ease of purification compensates for the reduction in yield to 57%. The material obtained by concentration of the liquors recovered from recrystallization were found to consist mainly of *Z-***3** and *E*-**3** by ^1^H NMR spectroscopy. Purification of this material by chromatography gave an additional 11% yield of pure *Z-***3** (3.7 g).

## Conclusion

An efficient continuous flow methodology has been developed for the three-step synthesis of α-thio-β-chloroacrylamide *Z-***3**, which has overcome the challenges to scale-up posed by the conventional batch preparation. This approach has yielded improvements in process safety, significantly reduced reaction times and increased product purity, obviating the need for chromatography. One process, preparation of α-thioamide **2** ultimately proved most efficient in batch, though the investigations performed in flow were critical to achieving the optimization. The easy access to synthetically useful amounts, afforded by a transfer to continuous processing, is expected to significantly increase the attractiveness of harnessing the enormous potential utility of α-thio-β-chloroacrylamides on a more widespread basis. Perhaps the most powerful outcome is the ability to control the β-chloroacrylamide cascade through continuous processing, leading to selective recovery of individual components of the reaction.

## Supporting Information

File 1 General information, experimental procedures, analytical data and copies of NMR spectra of all compounds.
