# Supplementary material for: Development of a continuous process for α-thio-β-chloroacrylamide synthesis with enhanced control of a cascade transformation
Source: Beilstein J Org Chem. 2016 Nov 24;12:2511–22. doi: 10.3762/bjoc.12.246 (PMC5238589; doi:10.3762/bjoc.12.246)
Supplement: File 1 — General information, experimental procedures, analytical data and copies of NMR spectra of all compounds. [file Beilstein_J_Org_Chem-12-2511-s001.pdf]

Supporting Information

for

Development of a continuous process for α-thio-β-chloroacrylamide synthesis with enhanced control of a cascade transformation

Olga C. Dennehy[1], Valérie M. Y. Cacheux[1], Benjamin J. Deadman[†1], Denis Lynch[1], Stuart G. Collins*[1], Humphrey A. Moynihan*[1] and Anita R. Maguire*[2]

Address: [1] Department of Chemistry, Analytical and Biological Chemistry Research Facility, Synthesis and Solid State Pharmaceutical Centre, University College Cork, Cork, Ireland and [2] Department of Chemistry and School of Pharmacy, Analytical and Biological Chemistry Research Facility, Synthesis and Solid State Pharmaceutical Centre, University College Cork, Cork, Ireland

Email: Anita R. Maguire - a.maguire@ucc.ie;Humphrey A. Moynihan - H.Moynihan@ucc.ie; Stuart G. Collins - stuart.collins@ucc.ie

* Corresponding author

† Current address: Department of Chemistry, Imperial College London, UK

General information, experimental procedures, analytical data and copies of NMR spectra of all compounds

Contents

1. Experimental and characterization data……………..…………………………………S3

1.1. General procedures………..…………… ………………………………………..S3

1.2. Continuous flow setup……...………………………………..………………...S4

1.3. HPLC conditions for reaction analysis…..……...…………………………………...S6

1.4. Preparation of α-chloroamide **1**………..…………………..……………………......S8

1.5. Synthesis of α-thioamide **2**……………………..…….………………………….S9

1.6. Preparation α-thio-β-chloroacrylamide *Z*-**3** in continuous flow……...……..……..S12

1.7. References………………………………………………………………………S17

2. ^{1}H and ^{13}C NMR spectra for compounds **1–5** and **7**..S18

1. Experimental and Characterization Data

1.1 General procedures

Solvents were distilled prior to use as follows: dichloromethane was distilled from phosphorus pentoxide, ethyl acetate was distilled from potassium carbonate, hexane was distilled prior to use. Organic phases were dried using anhydrous magnesium sulfate. All commercial reagents were used without further purification unless otherwise stated.

^{1}H (300 MHz) and ^{13}C (75.5 MHz) NMR spectra were recorded on a Bruker Avance 300 MHz NMR spectrometer. ^{1}H (400 MHz) and ^{13}C (100.6 MHz) NMR spectra were recorded on a Bruker Avance 400 MHz NMR spectrometer. All spectra were recorded at 300 K in deuterated chloroform ($CDCl_3$) unless otherwise stated, using tetramethylsilane (TMS) as internal standard. Chemical shifts (δ_H and δ_C) are reported in parts per million (ppm) relative to TMS and coupling constants are expressed in hertz (Hz). Splitting patterns in ^{1}H spectra are designated as s (singlet), d (doublet), dd (doublet of doublets), ddd (doublet of doublet of doublets), t (triplet), q (quartet) and m (multiplet).

Infrared spectra were measured using a Perkin Elmer FTIR UATR2 spectrometer, or as potassium bromide discs (for solids) on a Perkin-Elmer Paragon 1000 FT-IR spectrometer. Wet flash column chromatography was carried out using Kieselgel silica gel 60, 0.040-0.063 mm (Merck). Thin layer chromatography (TLC) was carried out on pre-coated silica gel plates (Merck 60 PF254). Visualisation was achieved by UV (254 nm) light absorption.

Elemental analysis was carried out by Microanalysis Laboratory, National University of Ireland, Cork, using Perkin-Elmer 240 and Exeter Analytical CE440 elemental analysers. Low resolution mass spectra (LRMS) were recorded on a Waters Quattro Micro triple quadrupole instrument in electrospray ionization (ESI) mode using 50% acetonitrile-water containing 0.1% formic acid as eluent. High resolution (precise) mass spectra (HRMS) were recorded on a Waters LCT Premier Tof LC-MS instrument in electrosprayionization mode using 50% acetonitrile-water containing 0.1% formic acid as eluent. Samples prepared for either LRMS or HRMS by employing acetonitrile as solvent. Melting points were obtained using a uni-melt Thomas Hoover Capillary melting point apparatus and are uncorrected.

HPLC was performed on an Agilent Technologies 1120 Compact LC system (Agilent Technologies, Santa Clara, CA, USA) on Agilent Chemstation (Rev. B.04.03[52]) software

for data acquisition. Chromatography was carried out using a YMC-Pack ODS-A reverse phase column (250 mm x 4.6 mm, 5 μm) with a flow rate of 0.8 mL/min. Samples were injected (5 μL) by the autosampler and were detected by UV absorbance at 250 nm and by a 385-ELSD evaporative light scattering detector (LC-ELSD) (Agilent Technologies, Santa Clara, CA, USA). Nitrogen (99.995%) was used as the evaporation gas at a flow rate of 1.6 $L.min^{-1}$. The ELSD was operated with the nebulizer and evaporator temperatures at 40°C.

Experimental details for previously reported compounds **1, 2,** ***Z*-3, 4** and **5** prepared using batch reactions [1], have been included in this document to allow convenient comparison with the corresponding continuous process.

1.2 Continuous flow setup

All continuous processes were performed using either a Vapourtec R-Series flow reactor or a Vapourtec E-Series flow reactor.

The R-Series flow reactor consists of four piston pumps and up to four temperature controlled tubular reactors. To prepare the reactor for operation, pumps were purged with the solvent to be used in the reaction prior to use. All reaction tubing, coils, inlets and connections were also purged thoroughly in a similar manner.

Table S1: General specifications for R-Series continuous-flow reactor

General Specifications	
Material of tubing	PFA
Diameter of tubing	1 mm
Working flow rates	0.05 mL/min – 9.99 mL/min
Tubular reactor working volume	10 mL
Temperature range	-70 ^{0}C to 250 ^{0}C

The E-Series flow reactor consists of three peristaltic pumps and up to two temperature controlled tubular reactors. To prepare the reactor for operation pumps were, again, purged

with the solvent to be used in the reaction prior to use. All reaction tubing, coils, inlets and connections were also purged thoroughly in a similar manner.

Table S2: General specifications for E-Series continuous-flow reactor

General Specifications	
Material of tubing	PFA
Diameter of tubing	1 mm
Working flow rates	0.02 mL/min – 10.0 mL/min
Tubular reactor working volume	10 mL
Temperature range	-70 ^{0}C to 250 ^{0}C

1.3 HPLC conditions for reaction analysis

Due to the inherent complexity of the cascade process, the development of a robust HPLC method which would allow rapid quantitation of reaction products, intermediates and starting materials was considered to be highly advantageous for process optimization. A 40 min method employing isocratic 60:40 acetonitrile/water as the mobile phase gave resolution of the different reaction components (Table S3). The starting material **2**, key intermediates **4** and **5** and final product *Z*-**3** of the cascade could all be successfully identified by this method, using authentic isolated samples which were prepared in batch—in addition to the isomer *E*-**3** and the overchlorination products **6** and **7**. This method was used for optimization of the *β*-chloroacrylamide cascade in flow (Tables 2–5).

Table S3: Retention times for key components of the *α*-thio-*β*-chloroacrylamide cascade reaction by HPLC method[a].

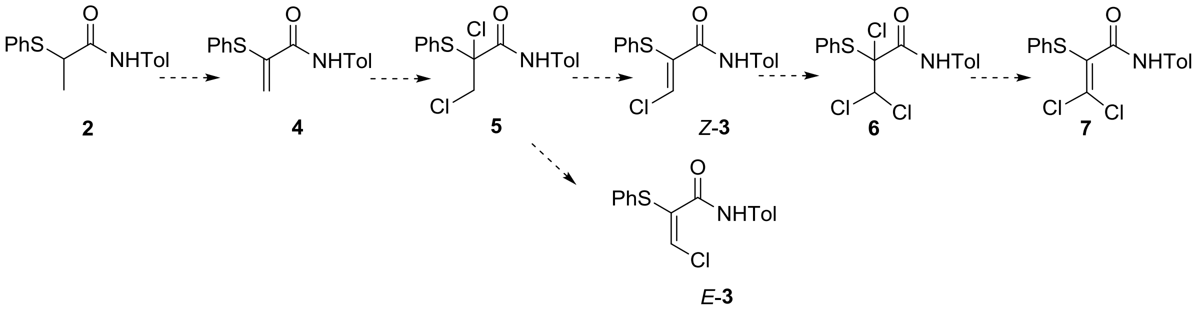

	**2**	**4**	**5**	***Z*-3**	***E*-3**	**6**[b]	**7**
**Retention times (min)**	12.2	14.9	26.3	20.0	13.1	33.0	18.3

[a]Chromatography was carried out using a YMC-Pack ODS-A reverse phase column (250 mm x 4.6 mm, 5 μm) with a flow rate of 0.8 mL/min. Samples were injected (5 μL) by the autosampler and were detected by UV absorbance at 250 nm and by a 385-ELSD evaporative light scattering detector (LC-ELSD). [b]The structure of this labile compound has been tentatively assigned based on comparison with related derivatives [1] by ^{1}H NMR spectroscopy of a mixture which also contained significant amounts of ***Z*-3, 5** and **7**.

In the case of Tables 2, 4 and 5, the product ratio was determined by weighting the peak areas generated against the relative response factors of the compounds under investigation at 250 nm.

Table S4: Relative response factors at 250 nm for key components of the *α*-thio-*β*-chloroacrylamide cascade reaction by HPLC method[a].

Component	Relative Response Factor[a] at 250 nm
**2**	1
**4**	4.41
Z-**3**	5.45
**5/6**	1[b]
E-**3**	5.93

[a]Determined using calibration curves. [b]As the compounds **5** and **6** could not be isolated, their relative response factors were estimated to give the same response as α-thioamide **2**, based on a similar level of conjugation.

A closely related 30 min method employing gradient 60:40–90:10 acetonitrile/water as mobile phase was used for analysis of the α-thioamide synthesis (Tables 1 and S5) affording resolution of the different reaction components (Table S5). The starting material **1**, final product **2** and diphenyl disulfide could all be successfully identified by this method, using authentic isolated samples prepared in batch. In order to accommodate running consecutive samples the following gradient was employed:

- 0-5 min - 60:40 MeCN/H_2O
- 5-15 min - 60:40 to 90:10 MeCN/H_2O (gradient)
- 15-20 min - 90:10 MeCN/H_2O
- 20-21 min - 90:10 to 60:40 MeCN/H_2O (gradient)
- 21-30 min 60:40

Table S5: Retention times for key components of the *α*-thioamide reaction by HPLC method[a].

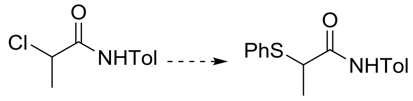

1 2

	1	2	PhSSPh
**Retention times (min)**	**7.1**	**10.9**	**18.8**

[a]Chromatography was carried out using a YMC-Pack ODS-A reverse phase column (250 mm x 4.6 mm, 5 μm) with a flow rate of 0.8 mL/min. Samples were injected (5 μL) by the autosampler and were detected by UV absorbance at 250 nm and by a 385-ELSD evaporative light scattering detector (LC-ELSD).

1.4 Preparation of α-chloroamide **1**

2-Chloropropionyl-*N*-(4-methylphenyl)propanamide (1) [1]

Original batch process

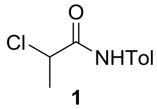

1

2-Chloropropionyl chloride (2.33 mL, 24.06 mmol) in dichloromethane (25 mL) was added dropwise over 20 min to a solution of *p*-toluidine (2.58 g, 24.06 mmol) and triethylamine (3.25 mL, 24.06 mmol) in dichloromethane (25 mL) at 0 °C, while stirring under nitrogen. Once the addition is completed, the reaction solution was removed from the ice bath and stirred at room temperature for 4 hours. Distilled water (40 mL) was added and the layers separated. The organic layer was washed with a saturated solution of sodium bicarbonate (2 x 30 mL), distilled water (40 mL) and brine (40 mL), dried, filtered and concentrated under reduced pressure to give the *amide* **1** (4.48 g, 94%) as a white solid not requiring further purification; m.p. 120–122 °C (Lit.[1] 118–120 °C); Found C, 60.75; H, 6.02; N, 7.03; Cl, 17.83; $C_{10}H_{12}NClO$ requires C, 60.77; H, 6.12; N, 7.09; Cl, 17.93%. ν_{max}/cm^{-1} (KBr) 3242.5 (NH), 1666.8 (CO); δ_H (300 MHz, $CDCl_3$) 1.83 [3H, d, *J* 7.4 Hz, C(3)H_3], 2.33 (3H, s, ArCH_3), 4.55 [1H, q, *J* 7.1 Hz,C(2)*H*], 7.16 (2H, d, *J* 8.3 Hz, Ar*H*), 7.43 (2H, d, *J* 8.4 Hz, Ar*H*), 8.22 (1H, br s, N*H*); δ_C (75.5 MHz, $CDCl_3$) 20.9 (Ar *C*H_3), 22.7 [*C*(3)H_3], 56.2 (*C*HCl), 120.1, 129.6 (aromatic *C*H), 134.4, 134.8 (aromatic *C*), 167.3 (*C*O).

Optimized continuous process

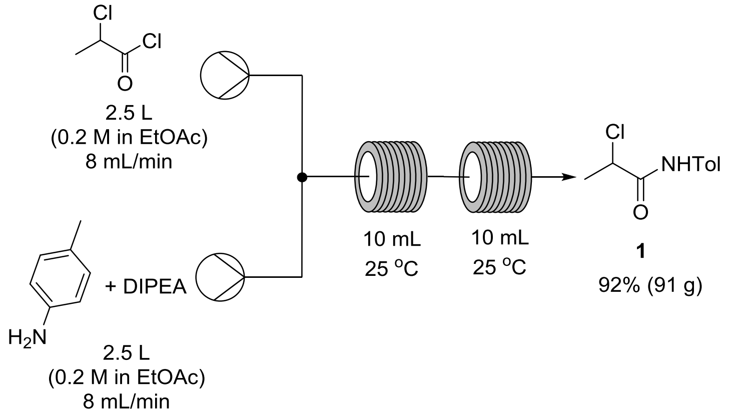

Scheme S1

A solution of 2-chloropropionyl chloride (65.4 g, 0.5 mol) in EtOAc (2.5 L) and a solution of *p*-toluidine (54.1 g, 0.5 mol) and diisopropylethylamine (65.3 g, 0.5 mol) in EtOAc (2.5 L) were prepared. The 2-chloropropionyl chloride solution was pumped (8 mL/min) into a T-

piece where it met the solution of *p*-toluidine and diisopropylethylamine (8 mL/min). The combined stream passed through two 10 mL reactor coils at 25 °C (2.5 min residence time). After exiting the reactor, the solution was collected and worked up in two portions. Each portion was washed with a saturated solution of sodium bicarbonate (1 L), distilled water (1 L) and brine (1 L), dried, filtered and concentrated under reduced pressure. The resulting solid was recrystallised from dichloromethane/hexane to give the *amide* (91.7 g, 93%) as a white solid, which demonstrated identical spectroscopic properties to those previously reported. The crude product can also be purified by recrystallization from EtOAc/heptane with a similar recovery.

1.5 Synthesis of α-thioamide **2**

2-(Phenylthio)-*N*-(4-methylphenyl)propanamide (2) [1]

Original batch process

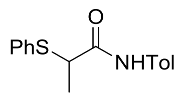

2

The title compound was prepared in a similar manner to that described previously [2]. Thiophenol (2.3 mL, 19.43 mmol) was added to a solution of freshly prepared sodium ethoxide [sodium (0.45 g, 19.43 mmol) in dry ethanol (50 mL)] at 0 °C while stirring under nitrogen. Immediately, a solution of 2-chloro-*N*-(4-methylphenyl)propanamide **(1)** (3.20 g, 16.19 mmol) in dry ethanol (40 mL) was added dropwise over 15 minutes to the reaction mixture. Following stirring for 17 hours at room temperature, the reaction was quenched on addition of water (50 mL) and dichloromethane (40 mL). The aqueous layer was extracted with dichloromethane (2 x 40 mL). Organic layers were washed with aqueous sodium hydroxide (1M, 2 x 50 mL), distilled water (80 mL) and brine (80 mL), dried and concentrated under reduced pressure. Purification of the crude product is by column chromatography on silica gel using hexane - ethyl acetate as eluent (gradient eluent 2–10% of ethyl acetate) to give pure *α-thioamide* **2** (3.42 g, 78%) as a white solid; mp 110–112 °C (Lit. [1] 112–113 °C); ν_{max}/cm^{-1} (KBr) 3289 (NH), 1663 (CO); δ_H (300 MHz, $CDCl_3$) 1.64 [3H, d, *J* 7.4 Hz,C(3)H_3], 2.31 (3H, s, ArCH_3), 3.90 [1H, q, *J* 7.3 Hz C(2)*H*], 7.11 (2H, d, *J* 8.1 Hz, Ar*H*), 7.22-7.41 (7H, m, Ar*H*), 8.36 (1H, br s, N*H*); δ_C (75.5 MHz, $CDCl_3$) 18.2 [*C*(3)H_3], 20.9 (ArCH_3), 47.9 [C(2)HS], 119.9, 127.7, 129.5, 129.6, 130.7 (aromatic *C*H), 133.4, 134.3, 134.9 (aromatic *C*), 168.9 (*C*O).

Continuous process – methanol as solvent

Due to the greater solubility of sodium chloride—a significant reaction by-product—in methanol compared to ethanol, methanol was investigated as a possible alternative solvent to ethanol with a view to enabling a flow process. A variety of temperature and reactant concentrations were examined (Table S6).

Table S6: Optimization of temperature, α-chloroamide **1** concentration and base for conversion of **1** to **2** in continuous mode[a] using MeOH as solvent

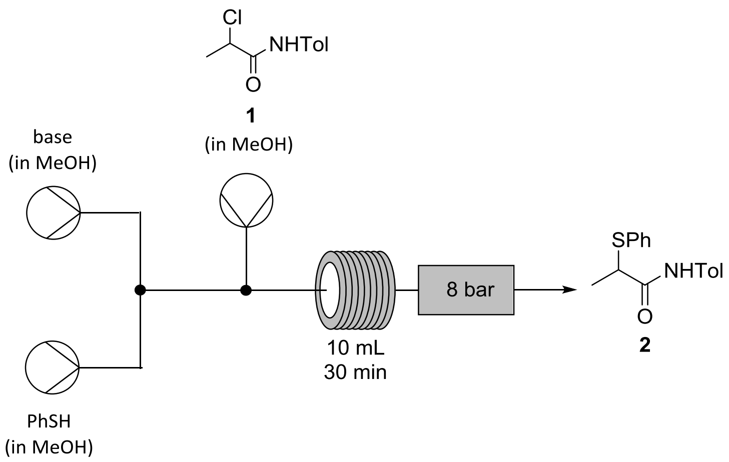

Entry	Temp. (°C)	α-Chloroamide 1 Conc.	Base (equiv.)	Product Ratio			
				2 (%[b])	1 (%[b])	PhSSPh (%[b])	Other[c] (%[b])
1	60	0.1 M	NaOMe[d] (1.2 eq.)	40.1	49.3	5.3	5.3
2	100	0.1 M	NaOMe[d] (1.2 eq.)	86.8	7.3	5.1	0.8
3	120	0.1 M	NaOMe[d] (1.2 eq.)	86.8	3.8	5.6	3.8
4	100	0.2 M	NaOMe[d] (1.2 eq.)	85.1	5.8	7.0	2.1
5	100	0.3 M[e]	NaOMe[d] (1.2 eq.)	85.6	7.1	3.1	4.2
6	100	0.1 M	NaOMe[e,f] (1.2 eq.)	35.6	32.0	9.1	23.3
7	100	0.1 M	Na_2CO_3 (1 eq.)	51.9	24.2	11.2	12.7
8	100	0.1 M	NaOH (10 eq.)	70.9	0	2.0	27.1

[a]General conditions: 1 equiv. α-chloroamide **1** (2 mL of solution in MeOH) was reacted with 1.4 equiv. PhSH and base (as a solution in MeOH) for 30 min. residence time. [b]Determined by HPLC analysis (peak area: see the Section 1.3) of samples taken directly from flow reactor as effluent solutions and diluted in MeCN prior to analysis. [c]Unisolated components, not present after workup. [d]NaOMe solution generated by reaction of sodium metal with dry MeOH. [e]Caused blockage of the back pressure regulator. [f]Commercial NaOMe.

As preparing sodium methoxide (from sodium metal and dry methanol) before each reaction is undesirable for large scale operation, alternative bases were also examined (Table S6). It is

also noteworthy, that commercial sodium methoxide performed poorly—compared to methoxide freshly prepared from sodium metal—due to problems with its solubility (entry 6, Table S6). Sodium carbonate was also found to give inferior results to those obtained with freshly prepared sodium methoxide (entry 7, Table S6). Use of 10 equivalents of sodium hydroxide, however, gave an acceptable yield of product, a reduced yield of diphenyl disulfide and no detected quantity of unreacted α-chloroamide **1** (entry 8, Table S6), the latter which had been a persistent impurity in all previous batches of **2**. While employing 10 equivalents of sodium hydroxide resulted in higher levels of unisolated impurities in the reaction medium (entry 8, Table S6), the crude product was otherwise found to contain only diphenyl disulfide as an impurity.

General procedure Table 1

Thiophenol solution (in ethanol) was pumped (0.11 mL/min) into a T-piece where it met aqueous sodium hydroxide solution (0.11 mL/min). The combined stream passed through a 32 cm tube before it met the *α*-chloroamide **1** solution (2 mL of a 0.1 M solution in EtOH) (0.11 mL/min) at a T-piece. This combined stream passed through a 10 mL convection flow coil reactor (5 min residence time). The product stream passed through a 50 cm tube and a back pressure regulator (8 bar) before exiting the reactor. Reactor effluents were collected in a round bottom flask and then concentrated under reduced pressure to remove the solvent.

Optimized batch process

Thiophenol (10.84 mL, 106.2 mmol) in ethanol (80 mL) was added to a solution of aqueous sodium hydroxide (0.8 M, 255 mL, 202.4 mmol). Immediately, a solution of 2-chloro-*N*-(4-methylphenyl)propanamide **(1)** (20.0 g, 101.8 mmol) in ethanol (255 mL) was added gradually over 15 minutes to the reaction mixture. Following heating under reflux for 1 hour, the reaction was cooled in an ice bath and was quenched by addition of water (100 mL). The solid precipitate was isolated by suction filtration. This gave pure *α-thioamide* **2** (24.44 g, 88%) as a white solid which exhibited identical spectroscopic properties to those previously reported.

1.6 Preparation of α-thio-β-chloroacrylamide *Z*-**3** in continuous flow

Original batch process

***N*-(4-Methylphenyl)-*Z*-3-chloro-2-(phenylthio)propenamide (*Z*-3)** [1]

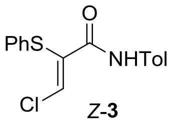

Z-**3**

N-Chlorosuccinimide (0.27 g, 32.01 mmol) was added to a solution of 2-(phenylthio)-*N*-(4-methylphenyl)propanamide (**2**) (0.28 g, 1.95 mmol) in toluene (6 mL). The flask was immediately immersed in an oil bath at 90 °C. Following stirring at 90 °C for 3 hours, the reaction mixture was cooled to 0 °C. The succinimide by-product was removed by filtration and the solvent was evaporated under reduced pressure. The crude product was purified by column chromatography on silica gel using hexane – ethyl acetate as eluent (gradient elution 2–10% ethyl acetate) gave the pure *β-chloroacrylamide Z*-**3** (0.26 g , 82 %) as a white solid; mp 106–108 °C (Lit. [1] 110–111 °C); ν_{max}/cm^{-1} 3336 (NH), 1650 (CO), 1518, 816; δ_H (300 MHz, $CDCl_3$) 2.29 (3H, s, ArC*H*$_3$), 7.09 (2H, d, *J* 8.4, Ar*H*), 7.20-7.31 (7H, m, Ar*H*), 8.04 (1H, s, C*H*Cl), 8.61 (1H, br s, N*H*); δ_C (100 MHz, $CDCl_3$) 20.8 (CH_3, Ar*C*H$_3$), 120.2, 127.3, 128.2, 129.5, 129.7 (9 x CH, aromatic *C*H), 130.7, 132.5, 134.5, 134.7 [4 x C, aromatic *C* and S*C*=], 140.7 (CH, *C*HCl), 160.3 (C, *C*=O); MS (ESI+): m/z 304 ($[M+H]^+$, 60 %) isotopic Cl pattern observed; 304, 306 (3:1 ^{35}Cl, ^{37}Cl).

***N*-4-Methylphenyl-2-(phenylthio)propenamide (4)** [1]

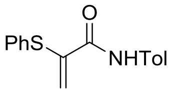

4

N-Chlorosuccinimide (0.16 g, 1.21 mmol) was added in one portion to a solution of 2-(phenylthio)-*N*-(4-methylphenyl)propanamide (**2**) (0.30 g, 1.10 mmol) in toluene (6 mL) at room temperature. Following stirring at room temperature for 24 h, the reaction mixture was filtered to remove the succinimide byproduct and the solvent was evaporated under reduced pressure. The crude product was purified by column chromatography on silica gel using a hexane – ethyl acetate eluent system (gradient elution 2–20% ethyl acetate) to give the *acrylamide* **4** (0.15 g, 49.5%) as a white solid; mp 101–102 °C; Found C, 71.11; H, 5.79; N, 5.11; S, 11.87; $C_{16}H_{15}NOS$ requires C; 71.34, H; 5.61, N; 5.20; S, 11.90%; ν_{max}/cm^{-1} (KBr) 3344.0 (NH), 1663.0 (CO); δ_H (300 MHz, $CDCl_3$) 2.29 (3H, s, ArC*H*$_3$), 6.13, 6.89 (2H, 2 x s, =C*H*$_2$), 7.09 (2H, d, *J* 8.1 Hz, Ar*H*), 7.18-7.36 (7H, m, Ar*H*), 8.53 (1H, s, N*H*); δ_C (75.5 MHz, $CDCl_3$) 20.9 (Ar*C*H$_3$), 120.1, 127.4, 128.9, 129.5, 129.6 (aromatic *C*H), 132.9 (=*C*H$_2$), 133.5, 134.5, 134.7, 136.1 (aromatic *C* or S*C*=), 161.4 (*C*O).

<u>*N*-4-Methylphenyl-2,3-dichloro-2-(phenylthio)propanamide (5)</u> [1]

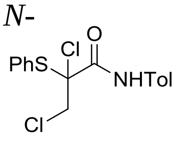

5

N-Chlorosuccinimide (0.32 g, 2.42 mmol) was added in one portion to a solution of *N*-(4'-methylphenyl)-2-(phenylthio)propanamide **3** (0.30 g, 1.11 mmol) in tetrachloromethane (6 mL) while stirring at room temperature under nitrogen. The mixture was stirred for 10 min at room temperature then heated to 40 ^{o}C and stirred at this temperature for 17 h. Filtration and evaporation of the solvent from the filtrate under reduced pressure gave the crude product which by ^{1}H NMR analysis was found to contain at least 78% *dichloride* **5**, along with a minor amount of *α-thio*-β-*chloroacrylamide Z*-**3**. The *dichloride* **5** was characterised from sample which was a mixture with *Z*-**3**; ν_{max}/cm^{-1} 3334 (NH), 1673 (CO), 1516, 812; δ_H (400 MHz, $CDCl_3$) 2.34 (3H, s, ArCH_3), 3.95, 4.46 (2H, ABq, *J* 12.0, CH_2Cl), 7.01-7.72 (9H, m, 9 × Ar*H*), 8.06 (1H, s, N*H*); δ_C (100 MHz, $CDCl_3$) 29.5 (CH_3, Ar*C*H_3), 50.2 (CH_2, C(3)H_2), 96.1 (C, *C*Cl), 162.7 (C, *C*=O); Characteristic signals for the *α-thio-β-chloroacrylamide Z*-**3** were also present.

<u>*N*-(4′-Methylphenyl)-2,3,3-trichloro-2-(phenylthio)propanamide (6)</u>

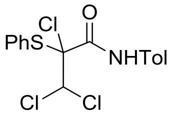

6

N-Chlorosuccinimide (0.17 g, 1.32 mmol) was added in one portion to a solution of *N*-(4′-methylphenyl)-*Z*-3-chloro-2-(phenylthio)propanamide **9** (0.20 g, 1.32 mmol) in tetrachloromethane (3 mL) while stirring at room temperature under nitrogen. The mixture was stirred at this temperature for 5 h. Filtration and evaporation of the solvent from the filtrate under reduced pressure gave the crude *trichloride* **6** which was characterised as a mixture with the *dichloride* **5**, the *acrylamide* **4**, the *dichloroacrylamide* **7** and the *α-thio*-β-*chloroacrylamide Z*-**3**; δ_H (400 MHz, $CDCl_3$) 2.65 (3H, s, CH_3), 6.56 (1H, s, C*H*), 6.89-7.29 (9H, m, Ar*H*), 7.79 (1H, s, N*H*), 7.38-7.42 (3H, m, Ar*H*).

<u>*N*-(4-Methylphenyl)-3,3-dichloro-2-(phenylthio)propenamide (7)</u>

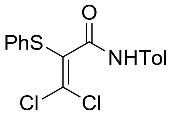

7

N-Chlorosuccinimide (0.22 g, 1.67 mmol) was added in one portion to a solution of *N*-(4′-methylphenyl)-*Z*-3-chloro-2-(phenylthio)propanamide **9** (0.20 g, 0.65 mmol) in toluene (6 mL). The flask was immediately immersed in an oil bath at 110 ^{o}C while stirring, under nitrogen, and maintained under refux for 5 h. Then the reaction mixture was cooled to 0 ^{o}C. The succinimide by-product was removed by filtration and the solvent was evaporated from the filtrate. The crude product was purified by successive attempts at column chromatography on silica gel using 1% ethyl acetate/hexane to

give the pure dichloroacrylamide **7** (0.043 g, 19.5 %) as a white solid; mp 128-130 ^oC; (Found C, 56.75; H, 3.99; N, 4.00; S, 9.16; Cl; 20.63. $C_{16}H_{13}Cl_2NOS$ requires C, 56.82; H, 3.87; N, 4.14; S, 9.48; Cl, 20.96 %); ν_{max}/cm^{-1} 3244 (NH), 2920, 1647 (CO), 1508, 812, 748, 689; δ_H (400 MHz, $CDCl_3$) 2.27 (3H, s, C*H*$_3$), 7.02-7.10 (4H, m, apparent q, 4 × Ar*H*), 7.28-7.34 (3H, m, 3 × Ar*H*), 7.40-7.51 (3H, overlapping signals: 2H, m, 2 x Ar*H*, and δ 7.44, br s, N*H*); δ_C (100.6 MHz, $CDCl_3$) 20.9 (CH_3, Ar*C*H_3), 120.4 (CH, aromatic *C*H), 125.2 (C, *C*SPh or *C*Cl_2), 128.9, 129.5 (CH, aromatic *C*H), 130.5 (C, aromatic *C*), 131.0 (C, *C*SPh or *C*Cl_2), 132.2 (CH, aromatic *C*H), 133.9, 135.0 (2 x C, aromatic *C*), 160.0 (C, *C*=O); MS (ESI+): m/z 340.1 [($C_{16}H_{15}{}^{37}Cl_2NOS$)+$H^+$], 338.1 [($C_{16}H_{15}{}^{35}Cl_2NOS$)+$H^+$]; HRMS (ESI+): Exact mass calculated for $C_{16}H_{14}Cl_2NOS$ $[M+H]^+$, 338.0173 Found 338.0169.

Only 4 of 5 possible aromatic CH carbon signals were observed at 100.6 MHz.

General procedure Table 2

α-Thioamide solution (0.01 M in toluene) was pumped into a T-piece where it met *N*-chlorosuccinimide solution (0.01 M in toluene). The combined stream was then passed through a convection flow coil reactor (10 mL), heated to 120 °C. The flow rates were adjusted to facilitate the residence time and the desired stoichiometry. The product stream passed through a back pressure regulator (8 bar) before exiting the reactor. Reactor effluents were collected in a round bottom flask and then concentrated under reduced pressure to remove the solvent. The components of the mixture were determined by HPLC analysis of samples taken directly from flow reactor as effluent solutions and diluted in MeCN prior to analysis.

General procedure Table 3

A solution of α-thioamide **2** (50 or 400 mmol) in solvent A (2 mL) was prepared. A solution of NCS (100 or 800 mmol) in solvent B (2 mL) was also prepared. The reagent solutions were injected into flowing streams (0.2 mL/min each) of solvent A or B. After the reagent solutions combined, they were passed into a convection flow coil reactor (10 mL) heated to 120 °C for 25 min before passing through a back pressure regulator (100 psi) and exiting the reactor. A sample of the reactor output was collected and the solvent removed by evaporation under reduced pressure. The sample was subsequently dissolved in $CDCl_3$ and analysed by

^{1}H NMR spectroscopy. The relative molar proportions of α-thioamide **2**, acrylamide **4**, dichloride **5** and β-chloroacrylamides *Z*-**3** and *E*-**3** were measured based on the integrals of their characteristic signals.

General procedure Table 4

α-Thioamide solution (4 mL, 0.2M in MeCN) was pumped into a T-piece where it met *N*-chlorosuccinimide solution (4 mL, 0.4M in MeCN). The combined stream was then passed through a convection flow coil reactor (10 mL). The product stream passed through a back pressure regulator (8 bar) before exiting the reactor. Reactor effluents were collected in a round bottom flask and then concentrated under reduced pressure to remove the solvent. The components of the mixture were determined by HPLC analysis of samples taken directly from flow reactor as effluent solutions and diluted in MeCN prior to analysis.

General procedure Table 5

α-Thioamide solution (4 mL, 0.2 M in MeCN) was pumped (2.5 mL/min) into a T-piece where it met *N*-chlorosuccinimide solution (in 4 mL MeCN) (2.5 mL/min). The combined stream was then passed through a convection flow coil reactor (10 mL), heated to 130 °C. The product stream passed through a back pressure regulator (8 bar) before exiting the reactor. Reactor effluents were collected in a round bottom flask and then concentrated under reduced pressure to remove the solvent. The components of the mixture were determined by HPLC analysis of samples taken directly from flow reactor as effluent solutions and diluted in MeCN prior to analysis.

<u>*N*-(4-Methylphenyl)-*E*-3-chloro-2-(phenylthio)propenamide (*E*-3)</u>

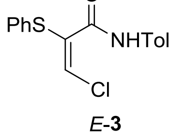

E-**3**

Following the general method (as applied to entry 5, Table 5), 50 mL of α-thioamide solution (0.2 M in MeCN) and 50 mL of *N*-chlorosuccinimide solution (0.4 M in MeCN) were reacted. The reactor effluents were collected in a round bottom flask and then concentrated under reduced pressure to remove the solvent. The crude product was then dissolved in toluene, and resulting solution was cooled to 0 °C and kept at this temperature for approximately 30 min, during which the succinimide by-

product precipitated and was removed by filtration. The filtrate was evaporated by reduced pressure and the resulting solid was analysed by ^{1}H NMR spectroscopy and found to contain a mixture of *Z*-**3** and *E*-**3** in a ratio of 86:14. to give of crude material The crude product material (3.0 g) was purified by column chromatography on silica gel using hexane – ethyl acetate as eluent (gradient elution 2–5 % ethyl acetate) to give the pure *E-isomer of the β-chloroacrylamide E*-**3** (102 mg, 3%) as a white solid; mp 100-102 °C;. Found: C, 63.76; H, 4.84; N, 4.34; S, 10.58; Cl, 11.72; $C_{16}H_{14}NOSCl$ requires C, 63.26; H, 4.65; N, 4.61; S, 10.55; Cl, 11.67%; ν_{max}/cm^{-1} (KBr) 3286.0 (NH), 1657.5 (CO); δ_H (300 MHz, $CDCl_3$) 2.29 (3H, s, ArC*H*$_3$), 6.96 (1H, s, C*H*Cl), 7.09 (2H, d, *J* 8.2 Hz, Ar*H*), 7.24-7.41 (7H, m, Ar*H*), 8.04 (1H, br s, N*H*); δ_C (75.5 MHz, $CDCl_3$) 20.9 (Ar*C*H$_3$), 120.1, 128.0 (aromatic *C*H), 128.4 (*C*HCl), 129.5, 129.6, 129.8 (aromatic *C*H), 131., 132.4, 134.4, 134.7 (quaternary aromatic *C* and S*C*=), 160.2 (CO); MS (ESI+): m/z 304 $(M+H)^+$ (94 %) isotopic Cl pattern observed; 304, 306 (3:1 ^{35}Cl, ^{37}Cl); HRMS (ESI+): Exact mass calcd for $C_{16}H_{15}NOSCl^{35}$ $(M+H)^+$ 304.0563.

Scale-up of optimized continuous process

A solution of α-thioamide (30.0 g, 0.11 mol) in MeCN (550 mL) and a solution of *N*-chlorosuccinimide (29.4 g, 0.22 mol) in MeCN (550 mL) were prepared.

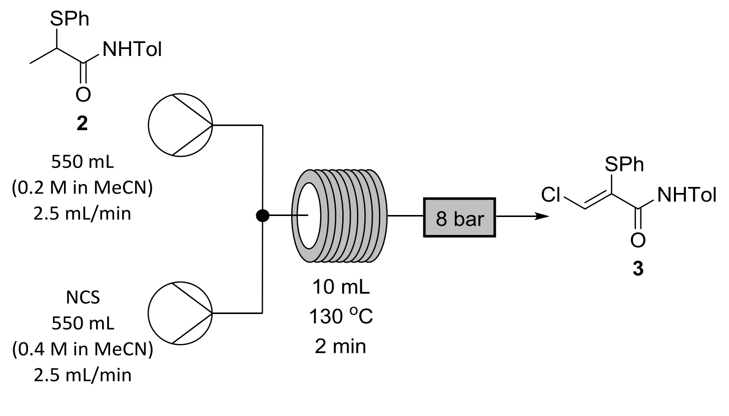

Scheme S2

The α-thioamide solution was pumped (2.5 mL/min) into a T-piece where it met the solution of NCS (2.5 mL/min). The combined stream passed through a 10 mL reactor coil at 130 °C (2.0 min residence time). The reactor output was collected and the solvent removed under reduced pressure. The crude product was then dissolved in toluene, and resulting solution was

cooled to 0 °C and kept at this temperature for approximately 30 min, during which the succinimide byproduct precipitated and was removed by filtration. The filtrate was evaporated by reduced pressure and the resulting solid was analysed by ^{1}H NMR spectroscopy and found to contain a mixture of *Z*-**3** and *E*-**3** in a ratio of 89:11. The crude product was purified by recrystallization from EtOAc/heptane to give the desired product *Z*-**3** as an off-white solid (19.28 g, 57%) which exhibited identical spectroscopic properties to those previously reported [1]. Concentration of the liquors gave crude material which was purified by wet flash chromatography using 1–5% EtOAc/hexane, affording an additional quantity of pure *Z*-**3** (3.68 g, 11%).

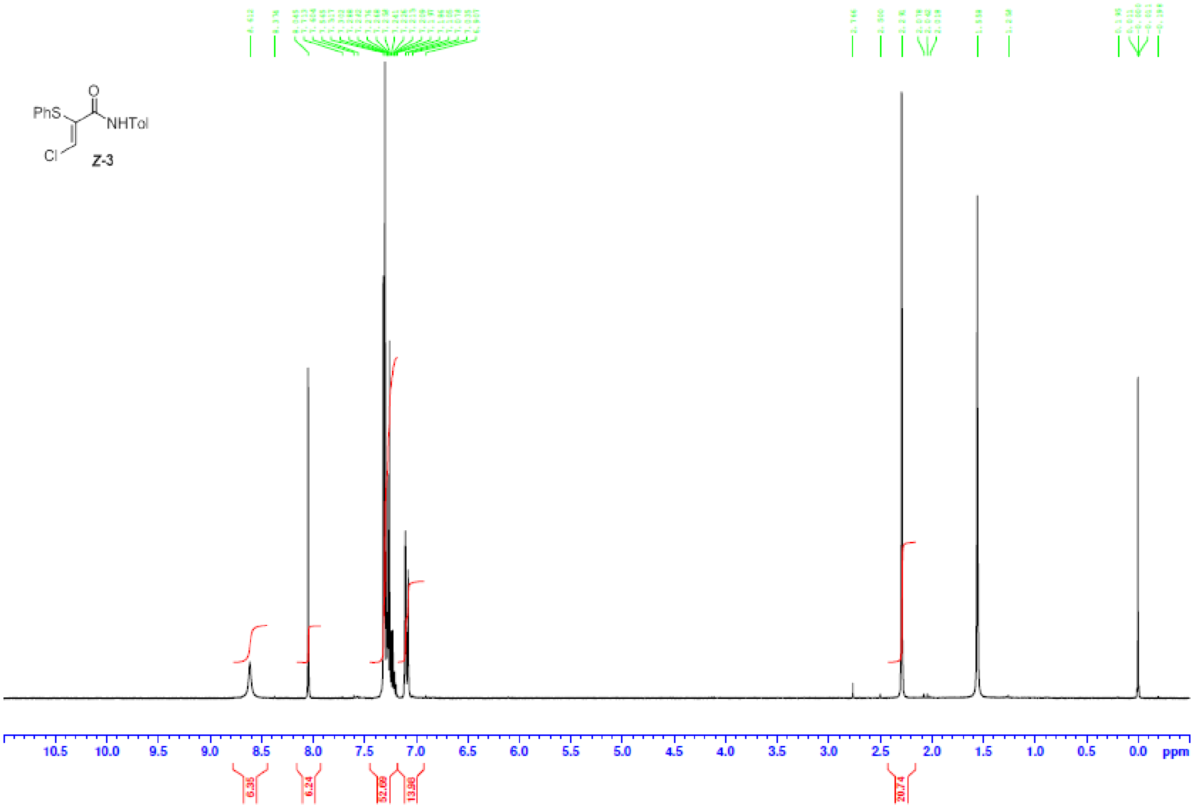

Figure S1.3: ^{1}H NMR spectrum of recrystallised *Z*-**3** from scaled-up continuous process.†

1.7 References

1. Murphy, M.; Lynch, D.; Schaeffer, M.; Kissane, M.; Chopra, J.; O’Brien, E.; Ford, A.; Ferguson, G.; Maguire, A. R. *Org. Biomol. Chem.* **2007**, *5*, 1228–1241.
2. Kissane, M.; Lawrence, S. E.; Maguire, A. R. *Tetrahedron Asymmetry* **2010**, *21*, 871–884.

2. Copies of ^{1}H and ^{13}C NMR Spectra for Compounds 1–5 and 7

†A peak for water was observed at 1.56 ppm.

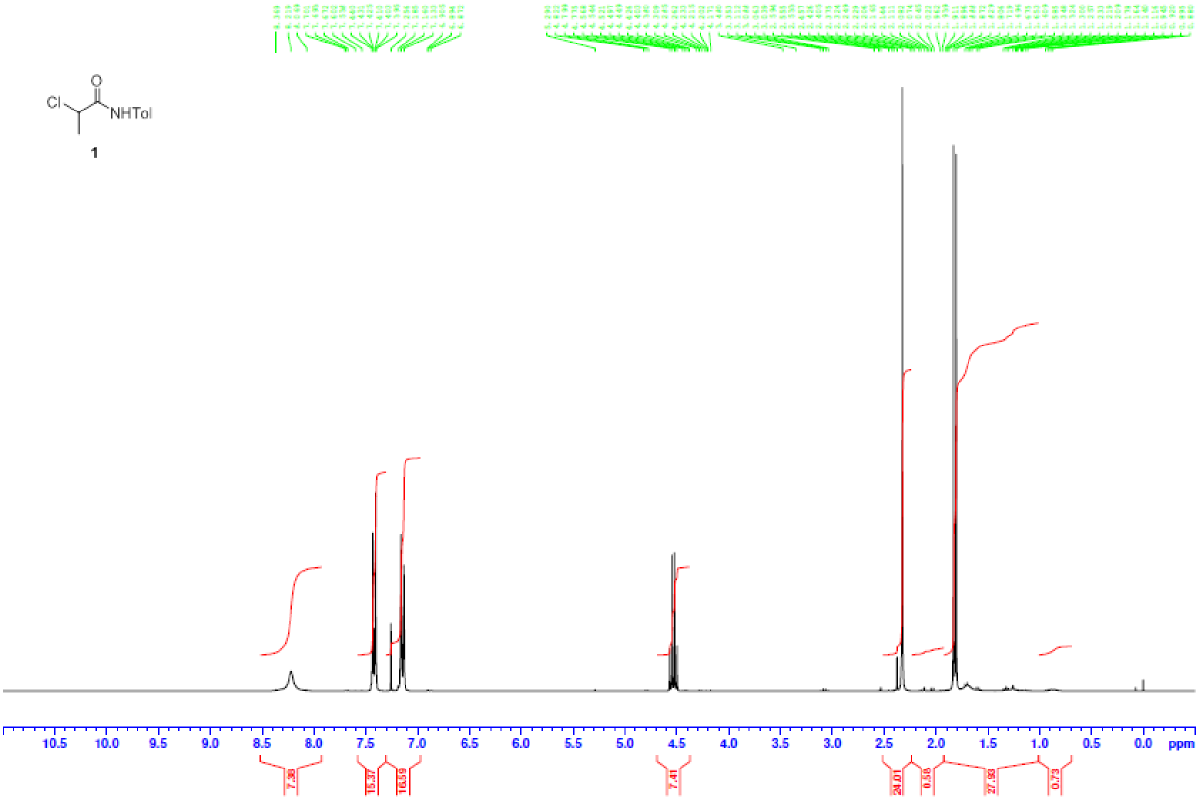

Figure S2.1: ^{1}H NMR (300 MHz, $CDCl_3$) spectrum of **1**.

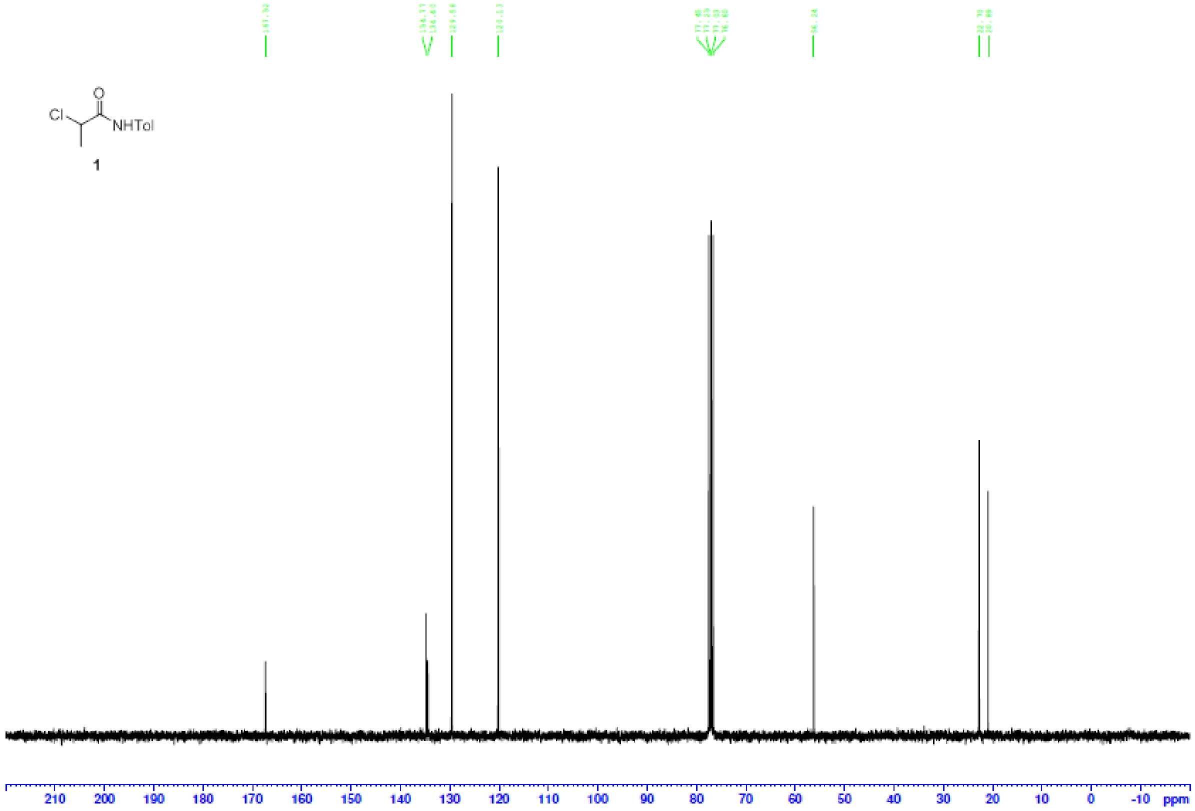

Figure S2.2: ^{13}C NMR (75.5 MHz, $CDCl_3$) spectrum of **1**.

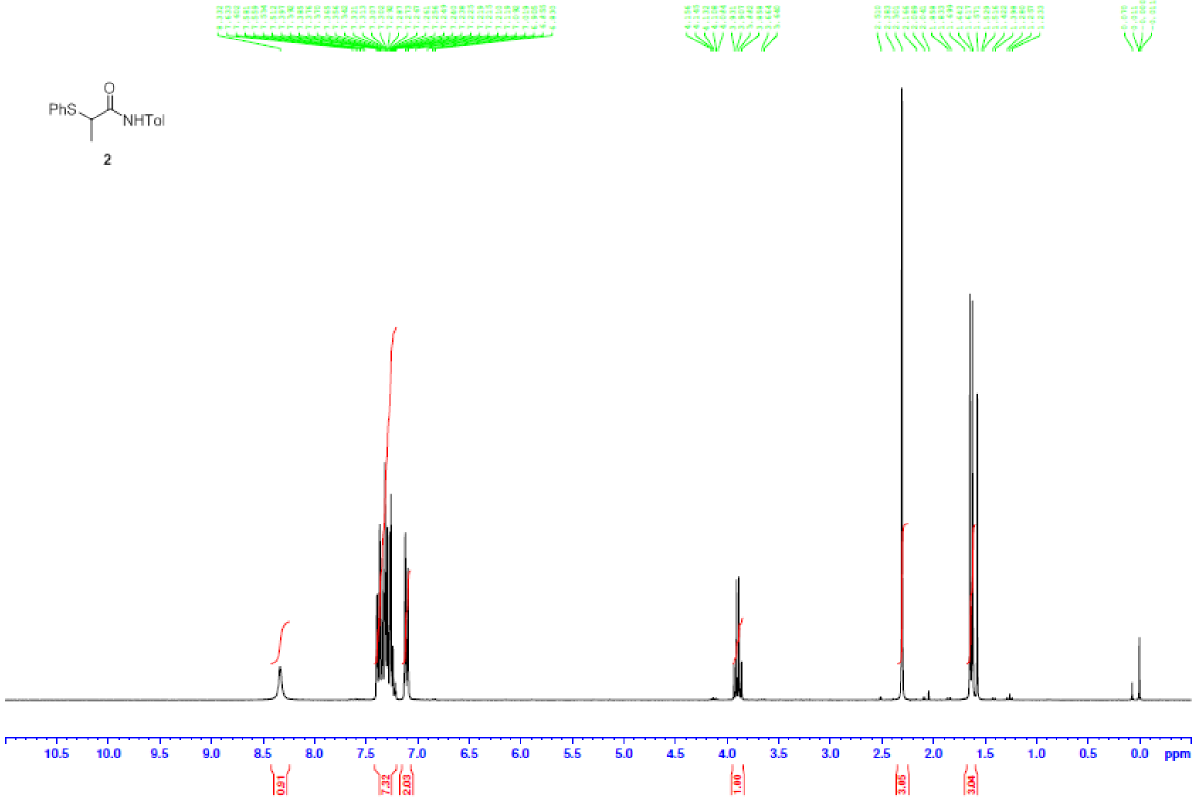

Figure S2.3: ^{1}H NMR (300 MHz, $CDCl_3$) spectrum of **2**.

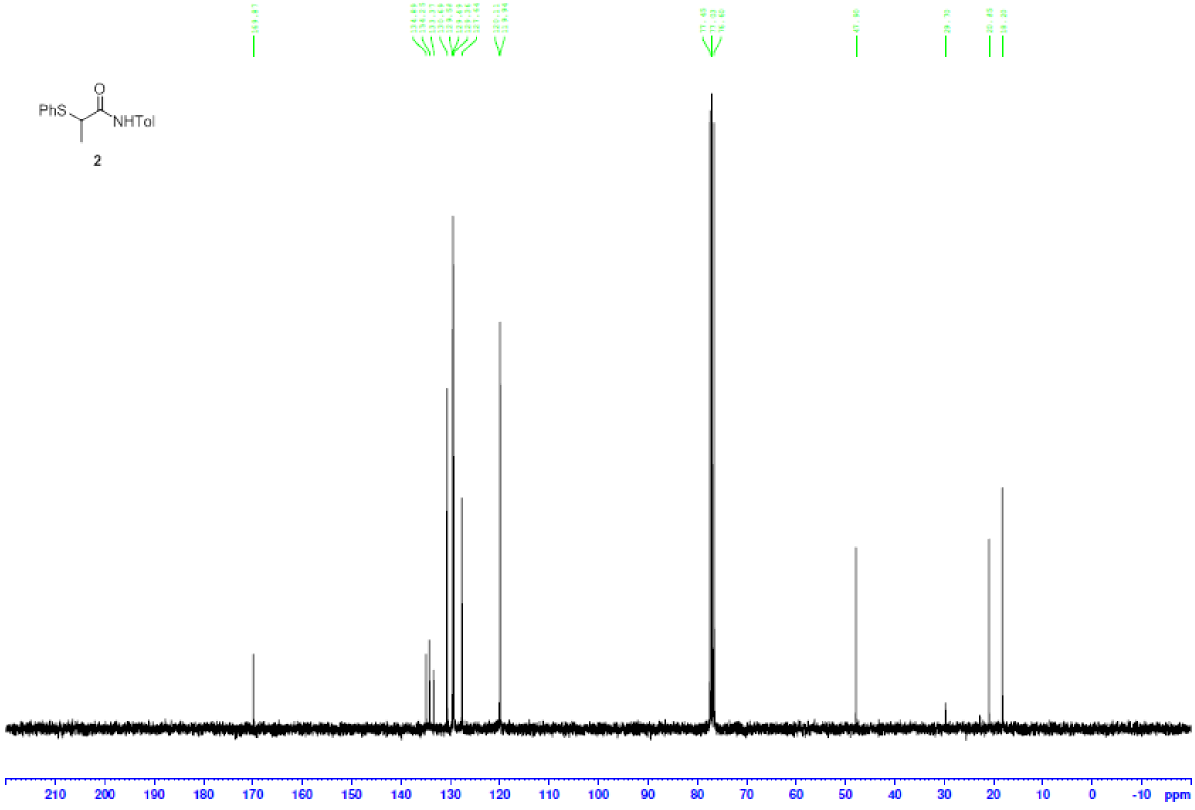

Figure S2.4: ^{13}C NMR (75.5 MHz, $CDCl_3$) spectrum of **2**.

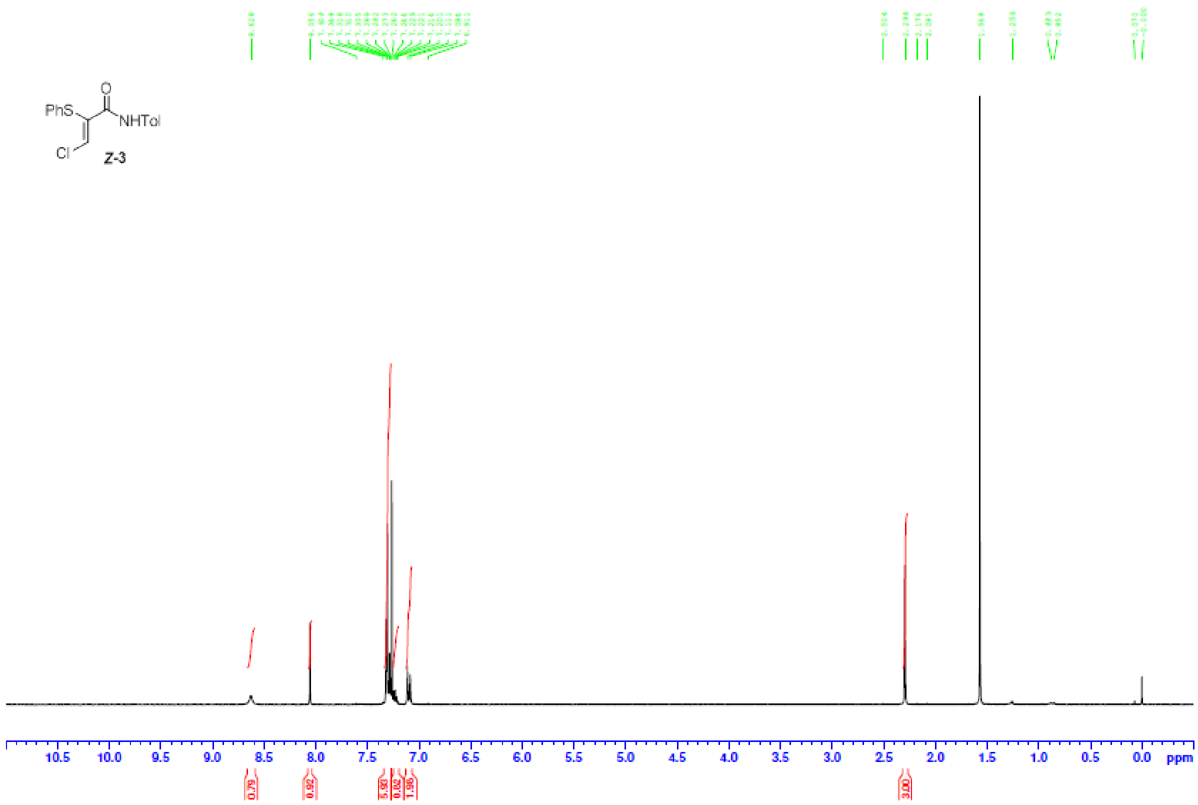

Figure S2.5: ^{1}H NMR (300 MHz, $CDCl_3$) spectrum of *Z*-**3**.

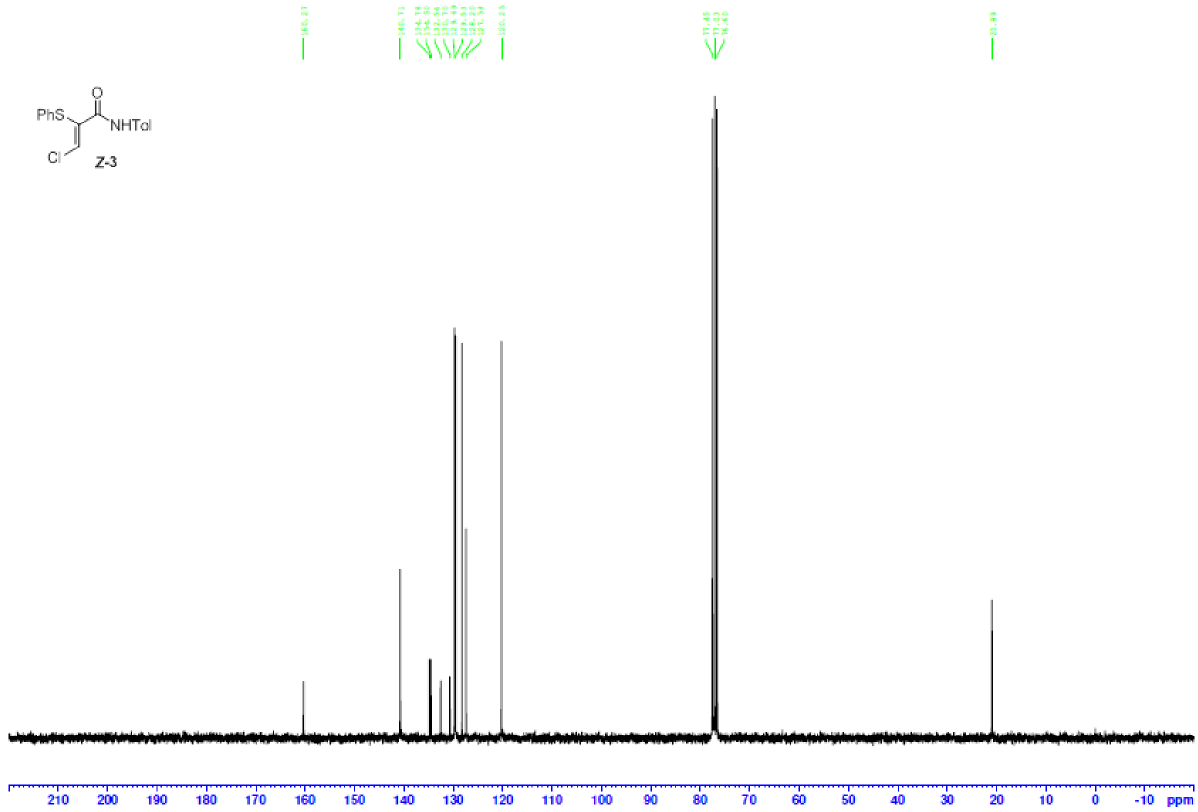

Figure S2.6: ^{13}C NMR (75.5 MHz, $CDCl_3$) spectrum of *Z*-**3**.

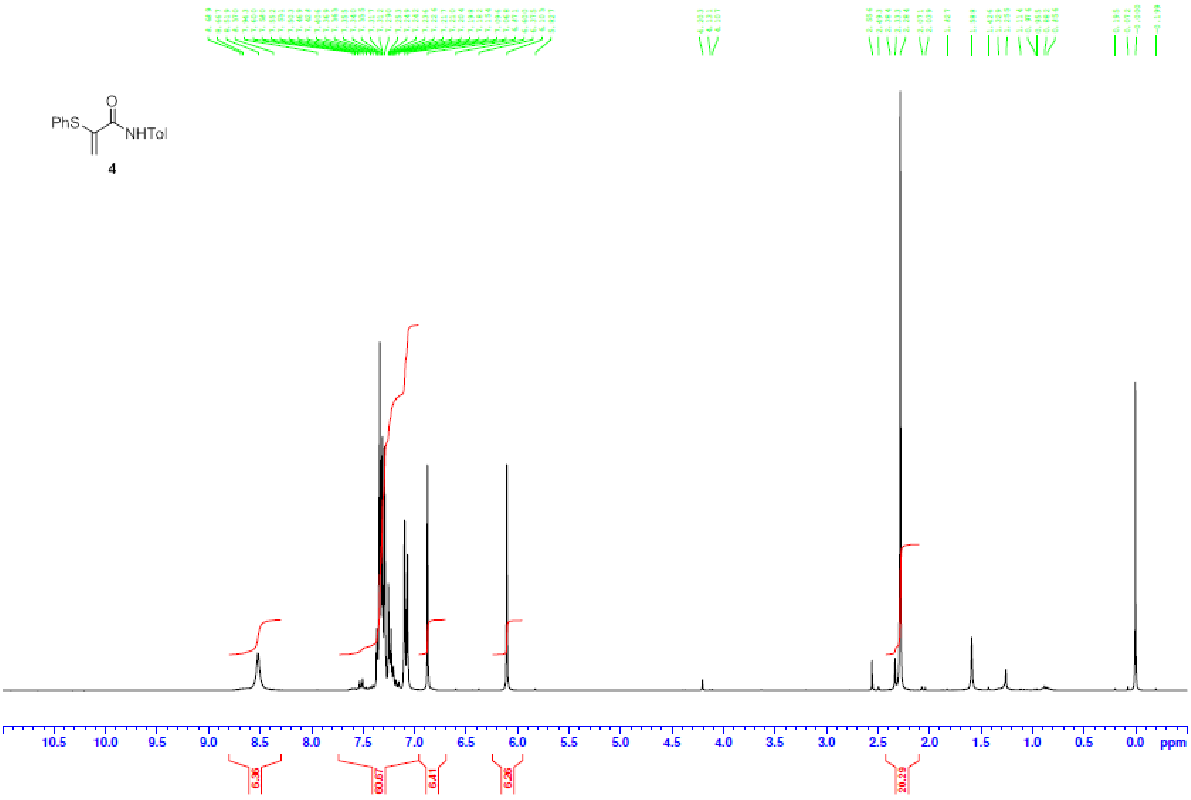

Figure S2.7: ^{1}H NMR (300 MHz, $CDCl_3$) spectrum of **4**.

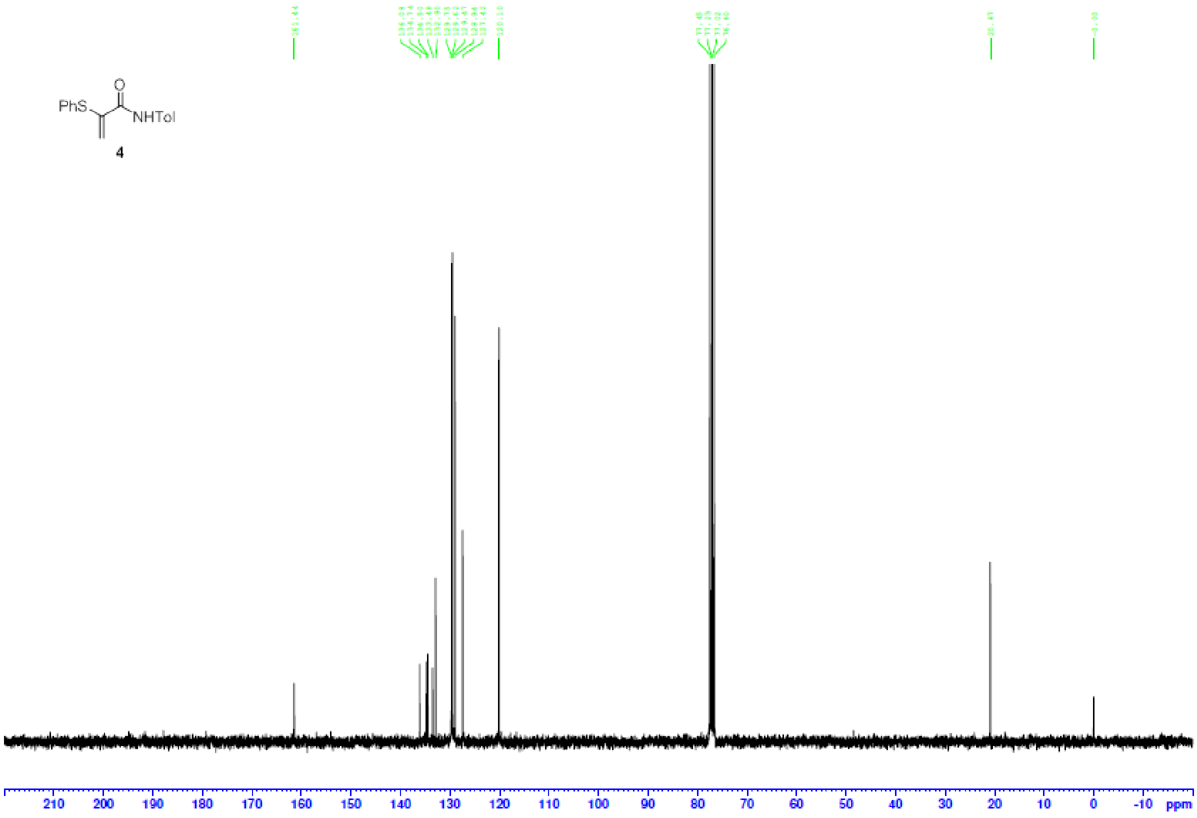

Figure S2.8: ^{13}C NMR (75.5 MHz, $CDCl_3$) spectrum of **4**.

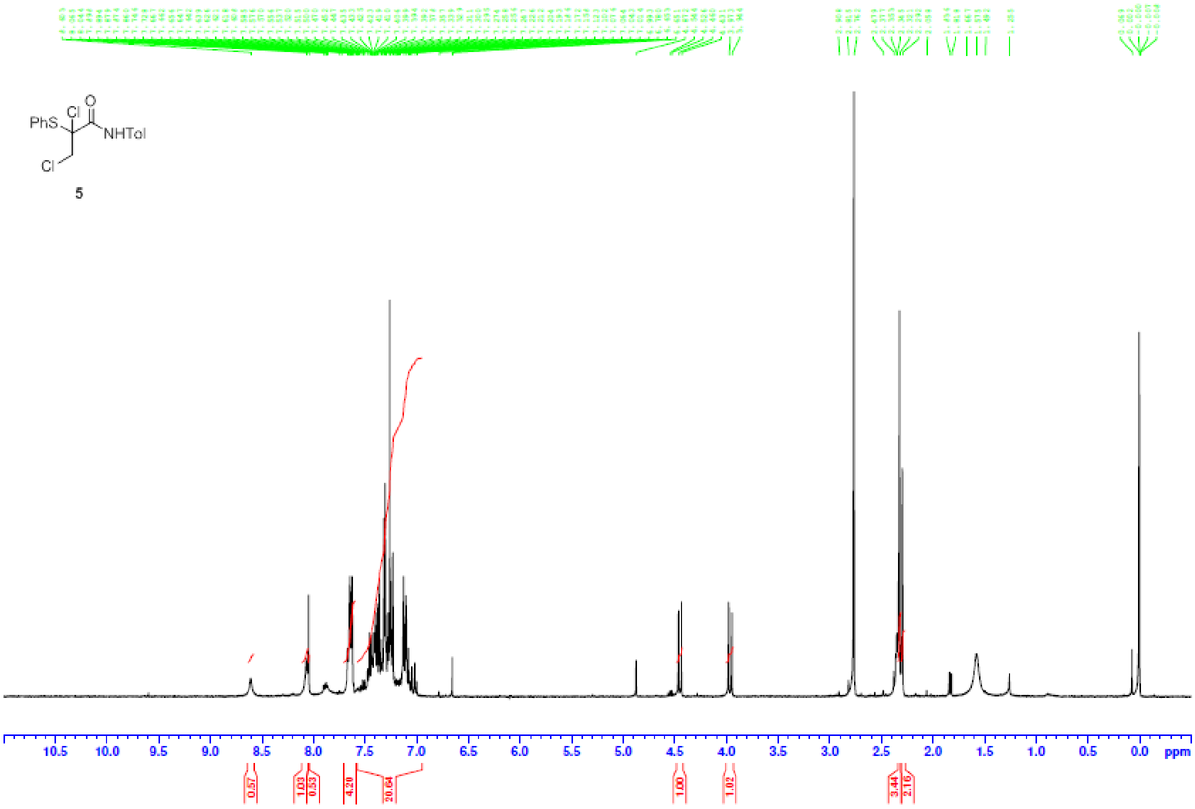

Figure S2.9: ^{1}H NMR (400 MHz, $CDCl_3$) spectrum of **5**‡

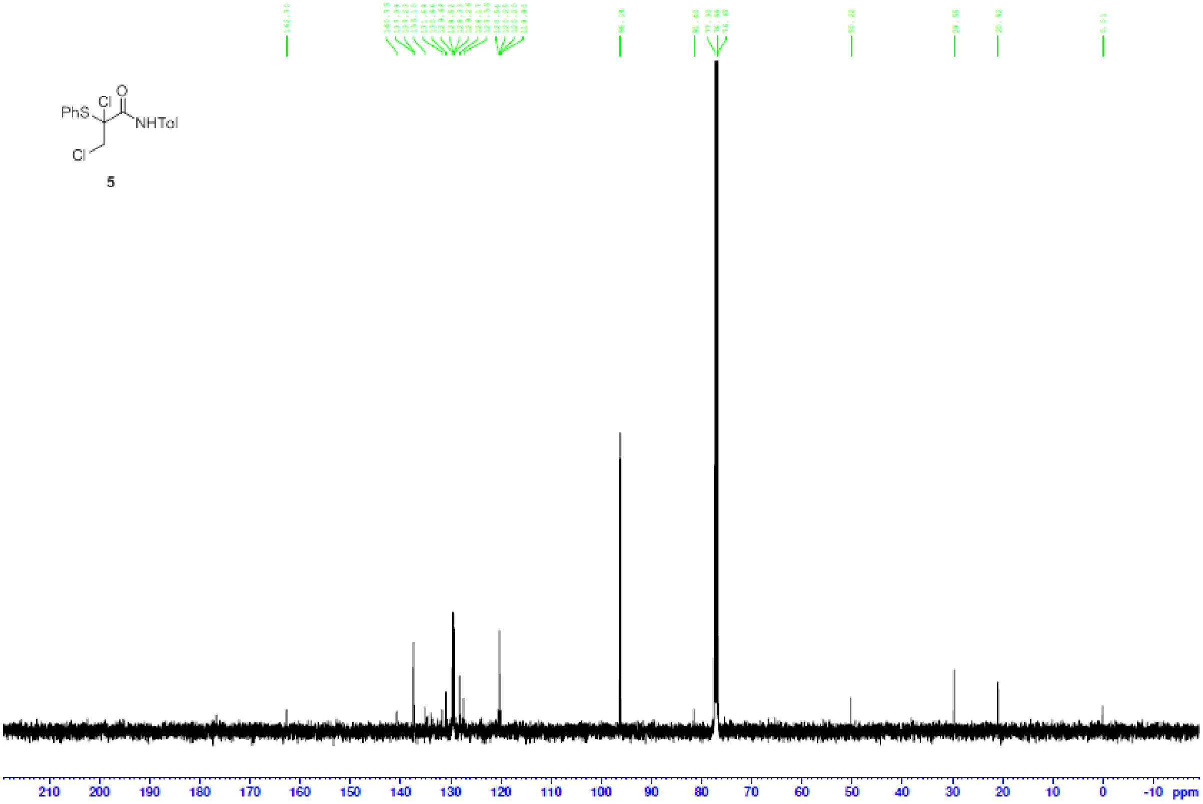

Figure S2.10: ^{13}C NMR (100.6 MHz, $CDCl_3$) spectrum of **5**‡

‡The dichloride **5** was characterised from sample which was a mixture containing at least 78% dichloride **5**, along with a minor amount of α-thio-β-chloroacrylamide *Z*-**3.**

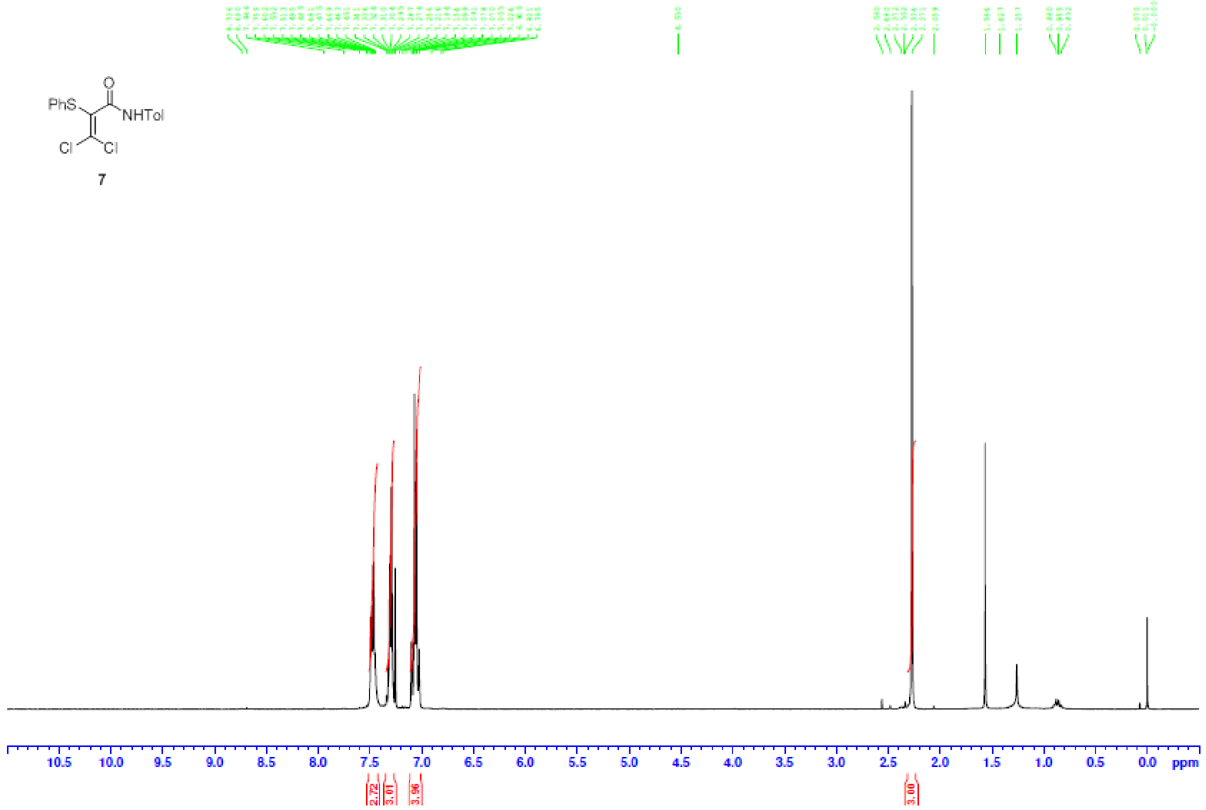

Figure S2.11: ^{1}H NMR (400 MHz, $CDCl_3$) spectrum of **7**.

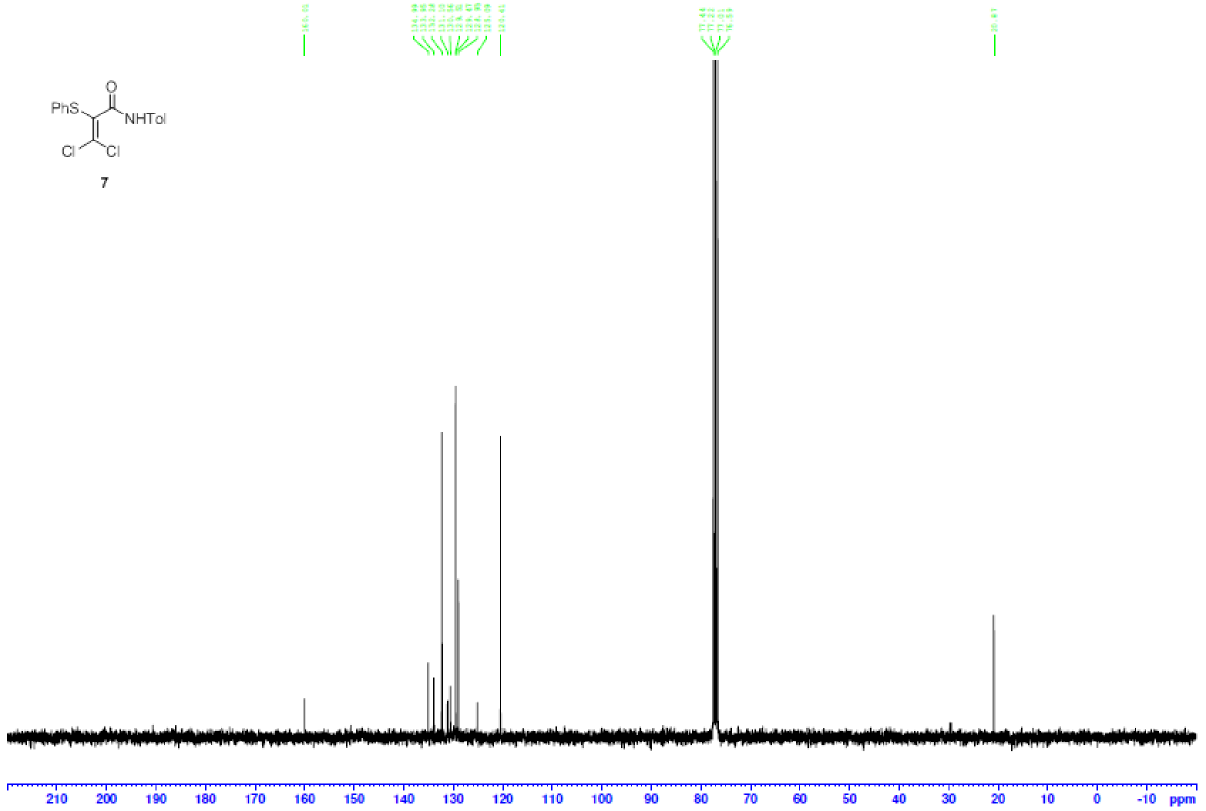

Figure S2.12: ^{13}C NMR (100.6 MHz, $CDCl_3$) spectrum of **7**.

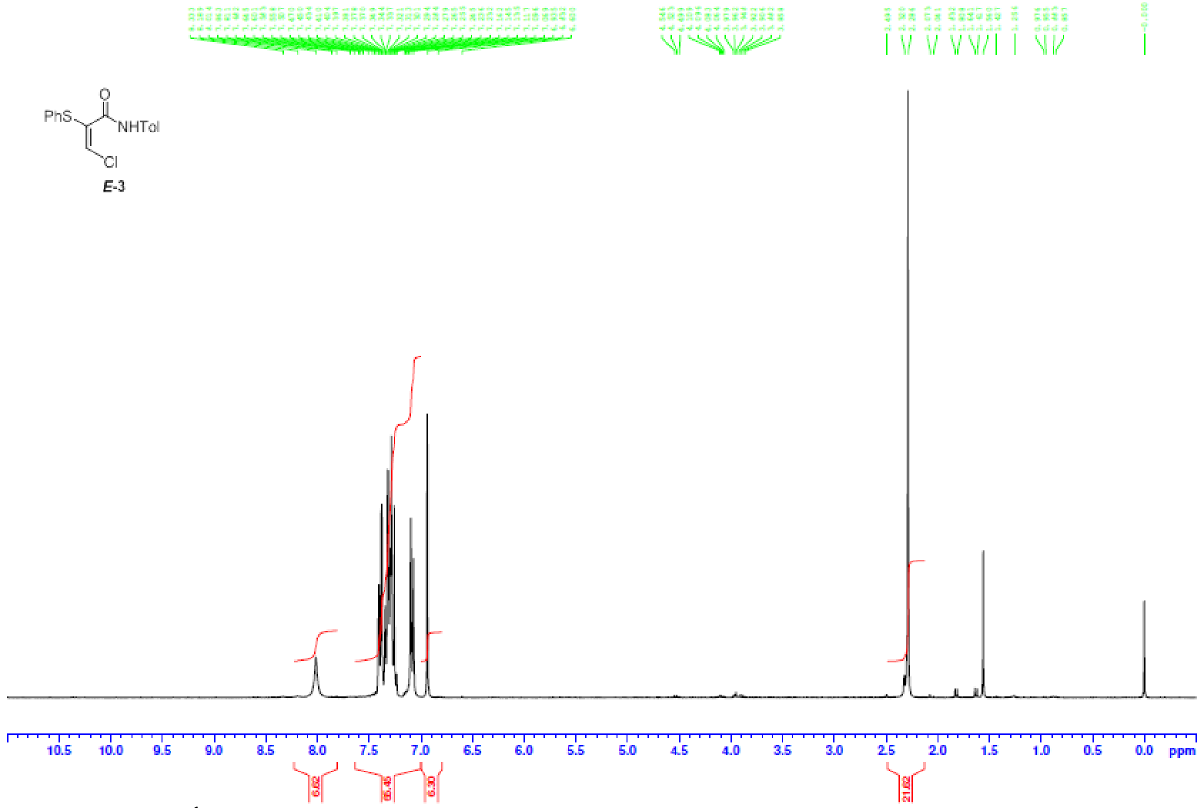

Figure S2.13: ^{1}H NMR (300MHz, $CDCl_3$) spectrum of *E*-**3**.

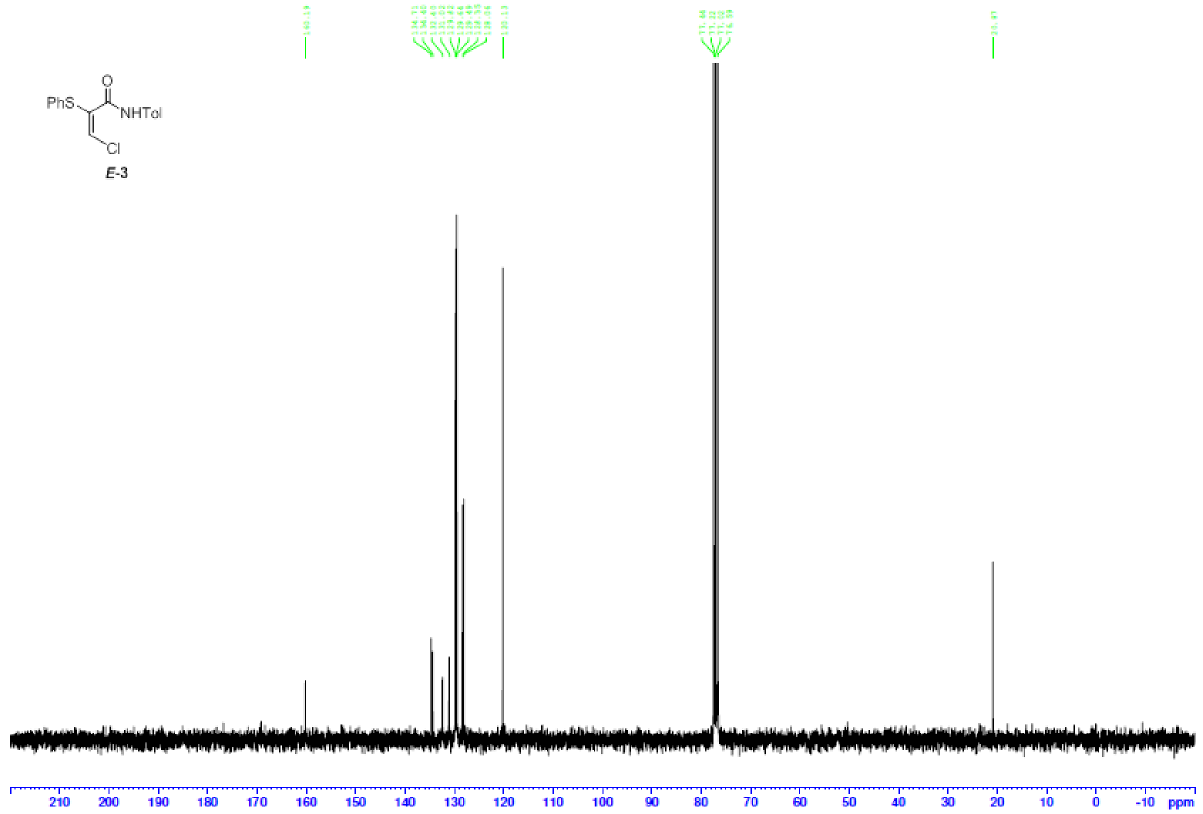

Figure S2.14: ^{13}C NMR (75.5 MHz, $CDCl_3$) spectrum of *E*-**3**.
